# Effects of Stress Coping Styles and Social Defeat on Zebrafish Behaviour and Brain Transcriptomics

**DOI:** 10.1007/s12264-025-01506-0

**Published:** 2025-09-22

**Authors:** Pavla Hubená, Lisa Benrejdal, David Brodin, Johanna Axling, Oly Sen Sarma, Peter Bergman, Svante Winberg

**Affiliations:** 1 Department of Medical Cell Biology, Behavioural Neuroendocrinology, Uppsala University, 751 23 Uppsala, Sweden; 2 Department of Biological and Environmental Sciences, University of Gothenburg, 405 30 Gothenburg, Sweden; 3 Department of Laboratory Medicine, Karolinska Institutet, 141 52 Huddinge, Sweden; 4 Bioinformatics and Expression Analysis Core Facility, Department of Medicine Huddinge, Karolinska Institutet, 141 52 Huddinge, Sweden; 5 Clinical Immunology and Transfusion Medicine, Karolinska University Hospital, 141 57 Huddinge, Sweden; 6 Department of Animal Biosciences, Swedish University of Agricultural Sciences, 750 07 Uppsala, Sweden

**Keywords:** Behavioural flexibility, Plasticity, Winning, Losing, Proactive, Reactive

## Abstract

Individuals with divergent personality traits corresponding to stress coping styles have been suggested to differ in behavioural and neural plasticity. We used a model of social defeat stress to assess the coping ability of wild zebrafish selectively bred for boldness/shyness. Behavioural tests were applied to assess parameters such as boldness/exploration, aggressiveness, and displacement behaviour. Gene expression changes in the brain were assessed *via* RNA sequencing. The main results show a strong effect of shyness and boldness phenotype on behaviour and the brain transcriptome. Fish of the shy line displayed significant behavioural differences, while the number of differentially-expressed genes remained low. In contrast, fish of the bold line exhibited a small effect on behaviour and pronounced changes in brain gene expression. This study highlights the importance of boldness phenotype and its influence on the response to social challenges at the behavioural and transcriptomic levels.

**Supplementary Information:**

The online version contains supplementary material available at 10.1007/s12264-025-01506-0.

## Introduction

Environmental instability increases the allostatic load on organisms, necessitating greater behavioural and physiological adaptation. Behavioural flexibility refers to the extent to which behaviour is influenced by environmental factors, and it is crucial for successful survival [1]. Neural plasticity, which involves biochemical changes and structural reorganisation, allows animals to operate beyond their typical behavioural phenotype [2]. Consequently, individuals in fluctuating environments are likely to benefit from the ability to quickly adapt, exploit new conditions, and experience minimal adverse effects from such adaptations [3]. Individuals confront these challenges with their correlated physiological and behavioural traits, which define their"stress coping style"and predispose them to varying degrees of resilience or vulnerability in specific situations [4–6]. While current research has concentrated on the adaptability of stress coping styles in non-social contexts, such as responses to novel environments, there is a notable lack of understanding regarding how individuals with different stress coping styles adapt to negative social circumstances. Following victories or defeats, transient changes in behaviour and brain gene expression occur alongside shifts in an individual’s social rank, reflecting biochemical switching processes [7, 8]. There is even less information available about the implications of such social experiences on the brain transcriptomics of individuals with varying stress coping styles, despite evidence linking defects in neural plasticity to the onset of neurodegenerative disorders [9].

The correlated suite of behavioural traits and physiological response to stress, which tends to remain consistent in an organism over time, is referred to as the"stress coping style". The"proactive"coping style is characterised by relatively higher levels of aggression, impulsivity, boldness or risk-taking, reliance on past experiences (feed-forward control), novelty-seeking, and significant frustration behaviour [1, 10]. This proactive phenotype is primarily distinguished from the"reactive"coping style at the physiological level by a lower cortisol response to stress (indicating reduced reactivity of the hypothalamic-pituitary-interrenal (HPI) axis; the hypothalamic-pituitary-adrenal axis in mammals) and elevated plasma catecholamine levels, suggesting increased sympathetic reactivity [10–13]. Further physiological differences have been noted among various stress coping strategies, including variations in antioxidant capacity, immunological markers, brain monoaminergic activity, and indicators of neural plasticity [10, 14, 15]. In contrast, the"reactive"coping style demonstrates an opposing set of behavioural and physiological responses compared to the proactive style. The type and intensity of behavioural reactions contribute to an individual's resilience or vulnerability to environmental stressors. For example, proactive individuals tend to resume feeding in novel environments more quickly thanks to their stress resilience, but struggle when they cannot depend on their memory to locate food [6]. As such, proactive fish seem to thrive in stable environments that allow for routine formation, while reactive fish may excel in variable, unpredictable settings [4, 6]. This difference may represent an indicator of environmentally stimulated behavioural flexibility. However, research into how behavioural flexibility is influenced by social factors has been limited to just one study [16]. The interaction of the stress coping style and social challenges and their potential impact on brain transcriptomics remains an area that requires further investigation.

Social rank in dominance hierarchies, which determines access to resources, is the result of social contests [17]. These contests are resolved through a specific set of ritualised agonistic behaviours unique to each species. In the case of zebrafish, encounters involve a variety of distinct agonistic behaviours, including displays, circling, striking, biting, chasing, retreating, fleeing, and freezing [18]. The contest ends with the establishment of a winner and a loser, with repeated interactions leading to the formation of a dominant/subordinate relationship. The outcomes of these contests have physiological effects on fish, influencing testosterone levels, HPI axis reactivity, vasotocin release, brain monoamine activation, and neural proliferation factors [7, 19–25]. These physiological differences underlie the behavioural change, with winners more likely to succeed in future contests—an effect commonly referred to as the'winner effect'. This can lead to aggressive behaviours where winners may initiate conflicts or'bully'the losers through biting and chasing [18, 24, 26]. Conversely, losers tend to exhibit a'loser effect', which is characterised by reduced aggressiveness, the display of submissive behaviours, and overall behavioural inhibition [18, 26]. Prior social experience is not only projected to further social performance, but there is also evidence that socially dominant chickadees (*Poecile gambeli*), meadow voles (*Microtus pennsylvanicus*), and mice (*Mus musculus*) tend to outperform their subordinate counterparts in non-social tasks like caching and spatial learning [27–29]. In fish, males of the African cichlid (*Astatotilapia burtoni*) that ascend in social rank have been observed to alter their behaviour in non-social contexts, becoming more cautious around novel objects and hesitant to enter reward areas [23]. Mismatched or incomplete adaptation of the behaviour relative to an individual’s social rank within the dominance hierarchy could potentially lead to an increased number of aggressive encounters and subsequent injuries [26, 30].

Selection for boldness and social interaction has consistently been shown to affect behavioural profiles [31] and brain gene expression [18, 32] in zebrafish, as well as in other species. Boldness has been identified as a stable behavioural trait that can predict the stress-coping style in fish [33]. Nevertheless, our understanding of the interplay between this behavioural trait and the social environment in shaping individual phenotypes remains insufficient.

The current study aimed to investigate the modulation of stress coping styles in response to varied social experiences. We aimed to estimate (i) the stability of the social behaviours (aggression, displacement behaviour) between bold and shy fish before and after social interactions; (ii) the differences in non-social behaviour (boldness/exploration, activity) displayed by bold and shy fish following these social experiences; and (iii) the impact of selective breeding and social experiences on gene expression in the brain. Zebrafish, which demonstrated specific stress coping strategies, were sourced from a selection programme based on bold and shy phenotypes, connected to different stress responses [34]. The bold and shy fish were then allowed to establish a dominance hierarchy through repeated dyadic contests, ensuring that equal numbers of bold and shy fish experienced both victories and defeats. Their behavioural profiles and gene expression were subsequently analysed. Gene expression was analyzed *via* bulk RNA sequencing of brain tissue samples, enabling comprehensive transcriptomic profiling across different fish lines following social exposure.

## Materials and Methods

In the current study, we used zebrafish males originating from an artificial selection programme focusing on differences in boldness and shyness as assessed by the novel tank diving test. Description of the selection programme is provided in the Supplementary files (SF-1). A total of 40 adult male zebrafish from the F2 generation were used, comprising 20 from the bold Line and 20 from the shy line. The fish had no prior experience with behavioural testing or any other procedure. The fish from the selection programme were housed in Aquaneering zebrafish racks (San Marcos, USA), in 6–9 L tanks. The temperature was 27.2 ± 0.3°C, pH 8.5, and the NH_3_/NH_4_^+^ was 0 in the housing racks. During the experiment, the fish were housed in 6 L tanks (29 cm × 20 cm × 15 cm) containing a filter (Eheim GmbH & Co., Deizisau, Germany) and a heater tempering the water to 27.9 ± 0.5°C. The NH_3_/NH_4_^+^ reached 0.7 ± 0.3 mg/L, and the pH was 8.5 throughout the whole experiment. Half of the tank volume (3 L) was exchanged once a day for aged, heated (28°C), and aerated water. The photoperiod was maintained at 14 h of Light and 10 h of darkness. The fish were fed twice a day with standard feed for zebrafish in laboratory conditions (Zebrafeed 400-600 mm, Sparos, Olhão, Portugal) and rotifers (*Brachionus* spp.).

### Experimental Design

**Fig. 1:**
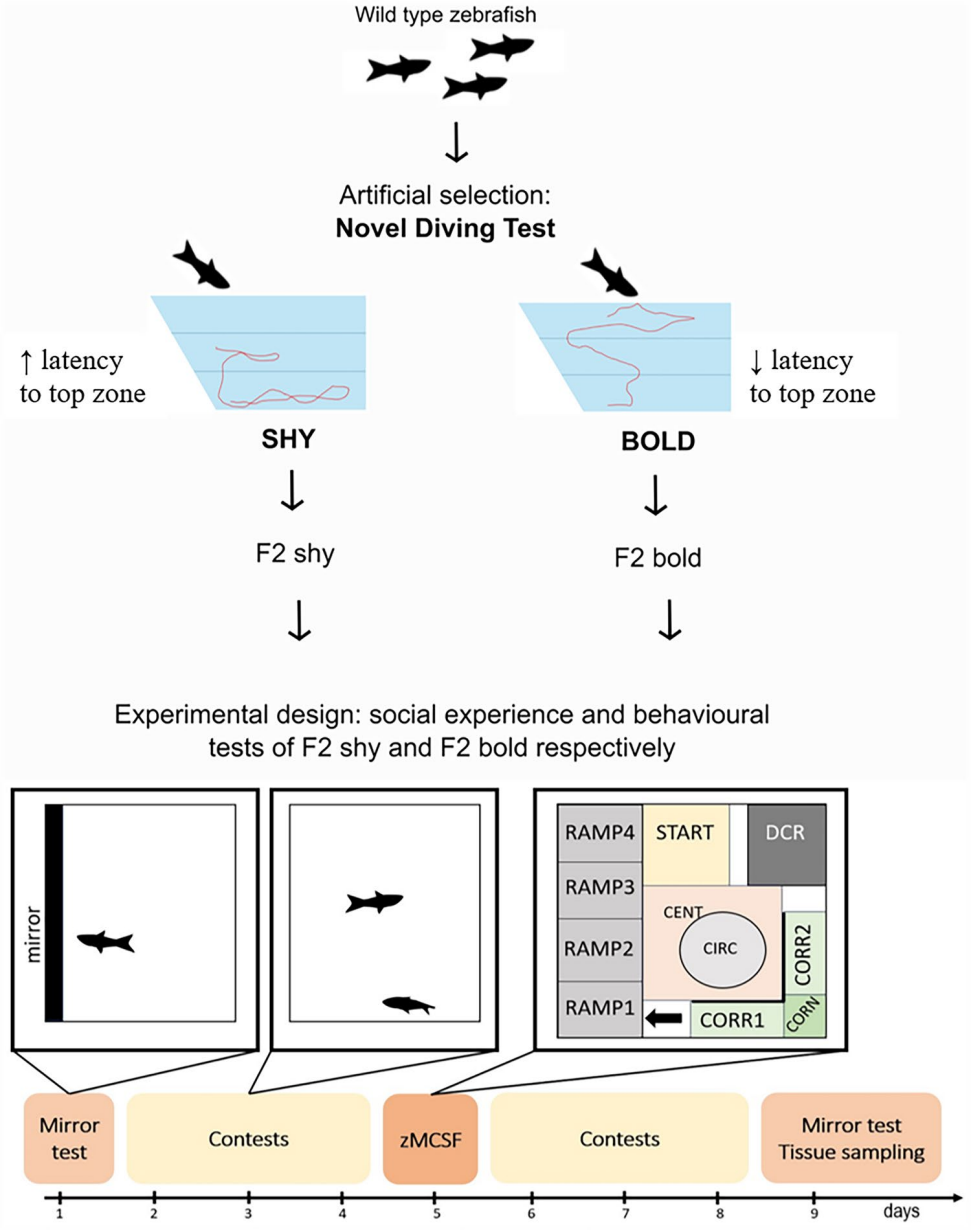
The experimental design of the artificially selected fish, including the mirror test assays, dyadic contests, and the zebrafish Multivariate Concentric Square-Field (zMCSF) test. The individual tests are visualized above the timeline. CENT, central zone; CIRC, circular zone; CORN, corner zone; CORR1, corridor 1; CORR2, corridor 2; DCR, dark corner with a roof; RAMP (1-4), inclined ramp with RAMP4 having the shallowest water depth; START, start zone. Unmarked zones belong to the REST zone. The black arrow indicates the entry to RAMP (1-4) zones

The timeline of the experiment is illustrated in Fig. 1. Four days prior to the beginning of the experiment, fish were anaesthetised by immersion in 200 mg/L benzocaine and were immediately transferred to experimental tanks, each tank holding a pair of fish, size-matched based on body weight (10% difference at maximum). The pairs consisted of familiar fish originating from the same selected line (bold or shy). The dorsal or the ventral side of the caudal fin was clipped to allow visual identification of individuals in each pair. The individuals in an experimental tank were divided by a liftable opaque barrier. The fish were allowed to acclimate to these conditions for three days. Following acclimation, each isolated fish was first allowed to fight with its mirror image in the mirror test (MT) in order to monitor differences in aggression and displacement behaviour prior to the social experience. Displacement behaviour is tested more in rodents (e.g., grooming) than in fish, but has been found to increase in response to stressful situations as a result of activation of the limbic system [35, 36]. During the MT, a mirror was slowly inserted into the compartment of individual fish, and its reaction was recorded for 10 min using a GigE camera. Following the MT, the partition separating pairs of fish was removed, and the fish were allowed to interact with their size-matched opponent. In order to establish a repeated winning or losing experience, the fish were allowed to interact for three days, 5 h per day. The outcome of the contests was visually assessed based on the subordinate behaviour (“tail drop”), initiation of fights by the winner, and occupation of the central part of the tank by the winning fish. In all pairs, a clear dominant and subordinate status was established after three days of social contest. After 3 days of repeated dyadic interaction, the fish were tested in the zebrafish Multivariate Concentric Square Field (zMCSF) test to monitor non-social behaviours. After this behavioural screening, the fish were returned to their experimental tanks and subjected to three more days of dyadic interaction with their size-matched opponent, 5 h per day, in order to re-establish the social experience. Following the dyadic contests, the fish were tested again in the MT in order to monitor the effects of social experience on aggression and displacement behaviour. After this final mirror test, the fish were immediately sacrificed in a 1000 mg/L benzocaine bath, and the brain was sampled within 3 min of euthanasia.

### Zebrafish MCSF Behavioural Test

A square opaque arena (30 cm × 30 cm × 25.8 cm) was filled with 8 L of aged, aerated, and pre-heated copper-free Uppsala municipal tap water (27.5 ± 0.3°C). Black plexiglass walls were placed at a 90° angle around a corner to create a corridor (zones CORR1, CORN, CORR2). A second corner was sheltered by a black plexiglass roof, which created a dark-roofed corner (DCR). An inclined transparent ramp was placed along the opposite wall (zones RAMP1, RAMP2, RAMP3, RAMP4) and provided a gradually reduced water depth. In order to prevent the preference of fish for specific zones based on external cues, the experiment was carried out using 4 arenas at the same time, and each arena was rotated at a 90° angle from the other arenas. These arenas were placed on top of an infrared backlight (Noldus, Wageningen, the Netherlands), and the trial was recorded by an infrared camera (Basler GigE) connected to the tracking system Ethovision XT 16 (Noldus, Wageningen, the Netherlands). The whole apparatus was enclosed in a separate behavioural room to prevent any disturbances. In this test, movement through the corridors was interpreted as locomotor activity and the movement through ramps and the open field area was translated as boldness/exploration, as described by Vossen *et al*. [37].

Two experimental tanks containing a random pair were placed into the behavioural room for five min for acclimation. Each individual was then netted from their compartment and placed into the START zone in their arena, which they were allowed to freely explore for 30 min. Their behaviour was recorded by the overhead camera and tracked in Ethovision XT 16. After the trial, the fish were transferred from the arena back into their original compartment, and the tank was placed back into its original location. The temperature in the arena was controlled before and after the trial.

### RNA Isolation and RNA Sequencing

A vertical section between the cerebellum and the optic tecta was made to divide the brain into forebrain and midbrain (FBMB) and hindbrain (HB). All samples were stored for 24 h in 500 μL of RNA*later* (commercially prepared solution) at 4 °C, after which they were relocated to −20°C until RNA isolation. A total of 12 pairs (*n =* 24 fish) were included in the transcriptomic analysis. The brain tissues were then transferred from the RNA*later* to 300 μL of lysis buffer (Qiagen, Inc., Germantown, USA) and homogenised using the TissueRuptor II (Qiagen, Inc.). RNA was isolated using the RNeasy Fibrous Tissue Mini Kit (Qiagen, Inc.) according to the manufacturer's protocol. Total RNA was subjected to quality control with Agilent Tapestation (Agilent, Santa Clara, USA) according to the manufacturer’s instructions. To construct libraries suitable for Illumina sequencing, the Illumina Stranded mRNA Prep (Illumina, San Diego, USA) Ligation preparation protocol was used, which included mRNA isolation, cDNA synthesis, Ligation of anchors, and amplification and indexing of the libraries. The yield and quality of the amplified libraries were analysed using Qubit by Thermo Fisher and the Agilent Tapestation. The indexed cDNA libraries were finally normalised and combined, and the pool was sequenced on two lanes of the Illumina Novaseq 6000 plus 10 B flowcell, 2× 150 bp, paired end mode, generating a total of 1937.52 M reads.

### Ethics Statement

The study was authorised by the Uppsala Animal Ethics Committee (permit 5.218-13393/2023). All procedures followed the guidelines of the Swedish Legislation on Animal Experimentation (Animal Welfare Act SFS 2018:1192) and the European Union Directive on the Protection of Animals Used for Scientific Purposes (Directive 2010/63/EU).

### Data Analysis

The ‘Line’ category contained fish with different behavioural phenotypes, bold (‘B’) or shy (‘S’). ‘Rank’ included divergent social experiences the fish received, losing (‘L’), or winning (‘W’). The ‘Zone’ category contained the 12 zones present in the zMCSF trial.

The ethological software BORIS [38] was used to score aggressive and displacement behaviour from the MT assay. The aggression was estimated based on the number, total duration, latency to first attack, and average duration of attacks. The average duration of an attack was calculated by dividing the total duration of the attack by the number of aggressive interactions. For aggressive behaviour to be recorded in zebrafish, they must have made physical contact with the mirror, which indicates overt aggression. Displacement behaviour was scored as the number, total and average duration, and latency to the first expression of the behaviour. Specifically, the fish exhibited a sudden compulsion to search for and bite at non-existent food, either from the bottom or the top of the tank. This was classified as displacement behaviour because food was not made available to the fish during the contest, and their reaction appeared as an abrupt behavioural shift that was inappropriate to the context of the threat posed by the mirror image.

Ethovision XT 16 software was used for the ethological evaluation of the recorded videos from the zMCSF test. Total distance moved (cm), average velocity (cm/s), and the immobility duration (s) were extracted for each arena. Total activity was calculated as the sum of all zone entries and was evaluated only during the whole-arena analysis. For zone-specific analysis, total distance moved (cm), average velocity (cm/s), the number of visits to each zone, latency to first entry to the zone (s), and cumulative duration in each zone (s) were extracted from the Ethovision software. Duration of stay in each zone was calculated by dividing the cumulative duration of stay in the zone by the duration of stay in the whole arena. Visit duration in each zone was calculated as the cumulative duration in each zone divided by the number of entries to the zone, and the total value was divided by the total duration of stay in the arena. The number of entries to zone (%) was calculated by subtracting the number of entries to zone from the total activity, and the result was divided by 100.

The transcriptomic data were analysed as follows: Basecalling and demultiplexing were applied using Illumina bcl2fastq (v2.20.0). Sequence data quality was assessed using FastQC (v0.11.8). Reads were aligned to the Ensembl *Danio rerio* GRCz11 reference genome using STAR (v2.7.9a).

Due to technical issues, four pairs of fish (*n =* 8) had to be excluded from the final analysis. The number of fish per selected line and social experience were equal (*n =* 8 per group; *n =* 32 in total).

### Statistical Analysis

The behavioural analysis was performed in R version 4.3.0 [39]. The packages ‘bestNormalize’ [40], ‘ggplot2’ [41], ‘emmeans’ [42], ‘lme4’ [43], ‘car’ [44], and ‘MASS’ [45] were used to analyse the behavioural dataset.

The aggressive and displacement behaviour was evaluated before the social experience for establishing differences between the behavioural phenotypes, i.e., the selected lines. Therefore, the Generalised Linear Mixed Effect Model (GLMM) with Poisson distribution and Line as fixed effect and individual as random effect was used to analyse the number of aggressive and displacement behaviours before the social experience. The latency to first attack or displacement behaviour, the total duration of attack or displacement behaviour, and the mean duration of attack or displacement behaviour were evaluated using one-way ANOVA with Line as the explanatory variable. The percentage change was used to analyse the effect of social experience, i.e., the change of behaviour from its original value. The percentage change of all aggressive and displacement behaviours was evaluated using two-way ANOVA with Line and Rank as explanatory variables. The zMCSF behavioural assay was analysed as behaviour in the whole arena and in specific zones. The total distance moved, average velocity, and the immobility duration in the whole arena in the zMCSF test were analysed using two-way ANOVA with Line and Rank as independent variables. The total activity in the zMCSF arena and the number of visits per zone were analysed using the GLMM with Negative Binomial distribution, as this model yielded a better fit (according to Akaike Information Criterion) than the Poisson distribution. The total distance moved, average velocity, the duration in each zone, visit duration, number of entries to zone (%), and latency to first entry were evaluated using the Linear Mixed Effect Models (LMM). Line and Rank were used as fixed factors, and the individual was applied as a random variable. Zone was added as a fixed factor for all zone-specific parameters in the zMCSF behavioural assay.

Normal distribution was assessed using the Shapiro-Wilk test of normality. The transformation method was recommended by the ‘bestNormalize’ package. Each parameter was transformed according to the recommended transformation if the normal distribution was not reached (for details, see Supplementary File SF-2). The emmeans function (‘emmeans’ package) with Bonferroni correction was used for pairwise comparison of factors in GLMMs, LMMs, and one- and two-way ANOVAs, and Bonferroni correction was used to adjust the *P*-value. Significant differences were evaluated based on *P* = 0.05. The weight of the fish was not significantly different between groups at the beginning of the experiment and was omitted from the analysis.

Counts for each gene were estimated using featureCounts (v1.5.1). The Bioconductor package DESeq2 (v.1.42.0) was used for sample group comparisons, generating log2 fold changes, Wald test *P*-values, and *P*-values adjusted for multiple testing (Benjamini-Hochberg method).

## Results

The objective of the experiment was to investigate differences in behaviour resulting from various social experiences and selected lines. To identify behavioural differences in aggression, displacement behaviour, boldness, and activity, an MT and the zMCSF test were applied (Fig. 1). In addition, the impact of selection for boldness and social experiences on the brain transcriptome was assessed through RNA sequencing of brain samples obtained after behavioural testing.

Generally, the stress coping style (see selection program in Supplementary File SF-1) produced more differences than the recent social experiences. This distinction was evident in the behavioural analyses (see Tables 1 and 2, Supplementary File SF-2) and the brain transcriptomics (Supplementary File SF-3).

**Table 1:** The initial differences between the selected lines in the aggressive and displacement behaviours (Trial 1) and the differences between groups in the percentage change of the parameters from the first to second trial, indicating the effect of social experience (Trial 1 - 2)

	Trial 1	Trial 1 - 2
**Aggression**		
Number of attacks	B > S**	BW < SW*
Latency to attack	NS	BL < SL*
Total duration of the attack	B > S*	NS
Average duration of attack	B > S*	NS
**Displaced behaviour**		
Number of displacement behaviours	NS	NS
Latency to displacement behaviour	NS	NS
Total duration of displacement	NS	NS
Average duration of displacement	NS	NS

B, bold selected line; BL, bold loser; BW, bold winner; NS, not significant; S, shy selected line; SL, shy loser; SW, shy winner. ***P < 0.001; **P = (0.01; 0.001); *P = (0.5; 0.1)

**Table 2:** Summary of zMCSF parameters for which there were significant differences between bold and shy winners and losers

Functional categories	Parameters	Selected lines	Social rank
Boldness/exploration	TDIS RAMP2	BL > SL*	
	TDIS RAMP3	BL > SL*	
	TDIS RAMP4	BW > SW***	
	AVEL CENT	BW > SW*	
	AVEL CIRC		SL > SW*
	DUR RAMP2	BL > SL*	
	DUR RAMP3	BL > SL*	
	DUR RAMP4	BL > SL* BW > SW***	
	VDUR RAMP4	BW > SW**	
	NVIS RAMP2	BL > SL*	
	NVIS RAMP3	BL > SL*	
	NVIS RAMP4	BL > SL* BW > SW***	
	NVIS (%) RAMP2	BL > SL*	
	NVIS (%) RAMP3	BL > SL*	
	NVIS (%) RAMP4	BW > SW***	
Locomotor activity	TDIS CORR2	BW > SW*	
	TDIS CORN	BW > SW*	
	DUR CORR2	BW > SW*	
	DUR CORN	BW > SW**	
	NVIS CORR2	BW > SW*	SL > SW*
	NVIS (%) CORR2	BW > SW**	SL > SW*
	NVIS (%) CORN	BW > SW*	
	LAT CORR1	BW < SW*	
	LAT CORR2	BW < SW*	
	LAT CORN	BW < SW*	BL > BW*

AVEL, average velocity (cm/s); DUR, duration in zone (s); LAT, latency to first entry (s); NVIS, number of visits to zone; NVIS (%), number of visits to zone in percentage; TDIS, total distance moved (cm); VDUR, visit duration (s)

### Aggression and Displacement Behaviour

There were significant differences in aggression and displacement behaviour before any social experience was induced, indicating a significant effect of the selected line (Table 1). Specifically, bold fish made significantly more attacks (GLMM: z = 2.783, *P =* 0.005), a longer duration of attacks (ANOVA: t_32, 30_ = 2.712, *P =* 0.011), and a longer average duration of attack (ANOVA: t_32, 30_ = 2.453, *P =* 0.02) than shy fish. The social experience was not considered in this analysis, as the fish had not been exposed to the social experience at that time and were isolated for three days prior to testing.

The percentage change reflects the variation in behaviour that occurred during the social experience. There were no significant changes from the baseline value (*P >* 0.05), but some groups differed in their percentage change. The percentage change of the number of attacks by bold winners was significantly lower than that of shy winners (ANOVA: t_32, 28_ = −2.632, *P =* 0.014). The percentage change of the latency to the first attack was significantly lower in bold losers than in shy losers (ANOVA: t_32, 28_ = −2.277, *P =* 0.031). The magnitude of the percentage change indicates how flexibly the fish reacted to altered social experience, indicating that both shy winners and losers are more flexible in their behaviour than their bold counterparts.

### Zebrafish Multivariate Concentric Square Field Test

When specific zones were not accounted for, the total distance moved, average velocity, total activity, and immobility were not significantly different between any selected line or social experience. Significant differences were found only when the zone factor was added to the calculation (see individual zones in Table 2). There were no differences in any parameter in the START, DCR, REST, and RAMP1 zones. However, there were differences in the CORR1/CORN/CORR2, RAMP2/RAMP3/RAMP4, and CENT/CIRC zones. For the full table, see Supplementary File SF-2.

Selected lines and social rank significantly affected the behaviour of fish in the CORR1/CORN/CORR2 zones. Bold winners entered CORR1 faster than shy winners (LMM: t_366, 288_ = 2.276, *P =* 0.024). Bold winners also entered CORN faster (LMM: t_366, 306_ = 2.480, *P =* 0.014), visited this zone more if compared in percentages (LMM: t_366, 306_ = 2.480, *P =* 0.014), spent longer in this zone (LMM: t_366, 260_ = 2.651, *P =* 0.009), and travelled longer distances in this zone (LMM: t_366, 171_ = 2.145, *P =* 0.033) than shy winners. Similarly, bold winners entered CORR2 faster (LMM: t_366, 288_ = 2.075, *P =* 0.039), visited this zone more (Negative Binomial GLMM: z = 2.331, *P =* 0.020; in percentages: LMM: t_366, 306_ = 2.904, *P =* 0.004), stayed there longer (LMM: t_366, 260_ = 2.272, *P =* 0.024), and travelled greater distances in this zone (LMM: t_366, 171_ = 2.533, *P =* 0.012) than shy winners. Social experience also significantly diversified the behaviour in the CORN and CORR2 zones. Specifically, bold losers entered the CORN zone later than bold winners (LMM: t_366, 290_ = 2.151, *P =* 0.032) and shy losers visited the CORR2 zone more than shy winners (Negative Binomial GLMM: z = 2.367, *P =* 0.018; in percentages: LMM: t_366,308_ = 2.162, *P =* 0.031).

There were significant differences in behaviour in RAMP (2–4) zones and CIRC/CENT zones between the selected lines, but no effect of social experiences. Specifically, bold losers visited RAMP2 more (Negative Binomial GLMM: z = 2.236, *P =* 0.025; in percentages: LMM: t_366, 307_ = 2.315, *P =* 0.021), spent a longer time (LMM: t_366, 271_ = 2.356, *P =* 0.019) and travelled longer distances there (LMM: t_366, 190_ = 2.447, *P =* 0.015) than shy losers. These differences were also significant in RAMP3 zone, as bold losers visited RAMP3 more (Negative Binomial GLMM: z = 2.480, *P =* 0.013; in percentages: LMM: t_366, 306_ = 2.090, *P =* 0.037), spent a longer time there (LMM: t_366, 265_ = 2.345, *P =* 0.020) and travelled longer distances in that zone (LMM: t_366, 180_ = 2.575, *P =* 0.011) than shy losers. Both bold winners and losers visited the RAMP4 zone more (Negative Binomial GLMM: W: z = 3.945, *P <*0.001; W (in %): LMM: t_366, 307_ = 4.029, *P <*0.001; L: z = 2.176, *P =* 0.030) and spent a longer time in this zone (LMM: W: t_366, 268_ = 4.055, *P <*0.001; L: t_366, 265_ = 2.158, *P =* 0.032) than shy winners and losers. However, only bold winners spent a significantly longer duration per visit in this zone (LMM: t_366, 250_ = 2.634, *P =* 0.009) and travelled longer distances through this zone (LMM: t_366, 182_ = 3.594, *P =* 0.004) than shy winners. This indicates that shy winners exhibited behaviour comparable to bold winners up to RAMP3 but avoided the RAMP4 zone more.

Zebrafish also differed significantly in average velocity in the CENT/CIRC zone, which was based on selected lines and social experience. Bold winners swam significantly faster through the CENT zone than shy winners (LMM: t_366, 111_ = 2.492, *P =* 0.014), but the average velocity only approached significance between bold and shy losers (LMM: t_366, 111_ = 1.967, *P =* 0.052). There was a difference between the selected lines depending on the social experience: shy losers moved significantly faster through the CIRC zone than shy winners (LMM: t_366, 126_ = 2.001, *P =* 0.048). Therefore, the social defeat seems to have caused an increase in swimming speed through the CIRC zone in shy, but not in bold fish.

### Transcriptional Changes in the Brain Related to Stress Coping Styles and Social Interactions

**Fig. 2:**
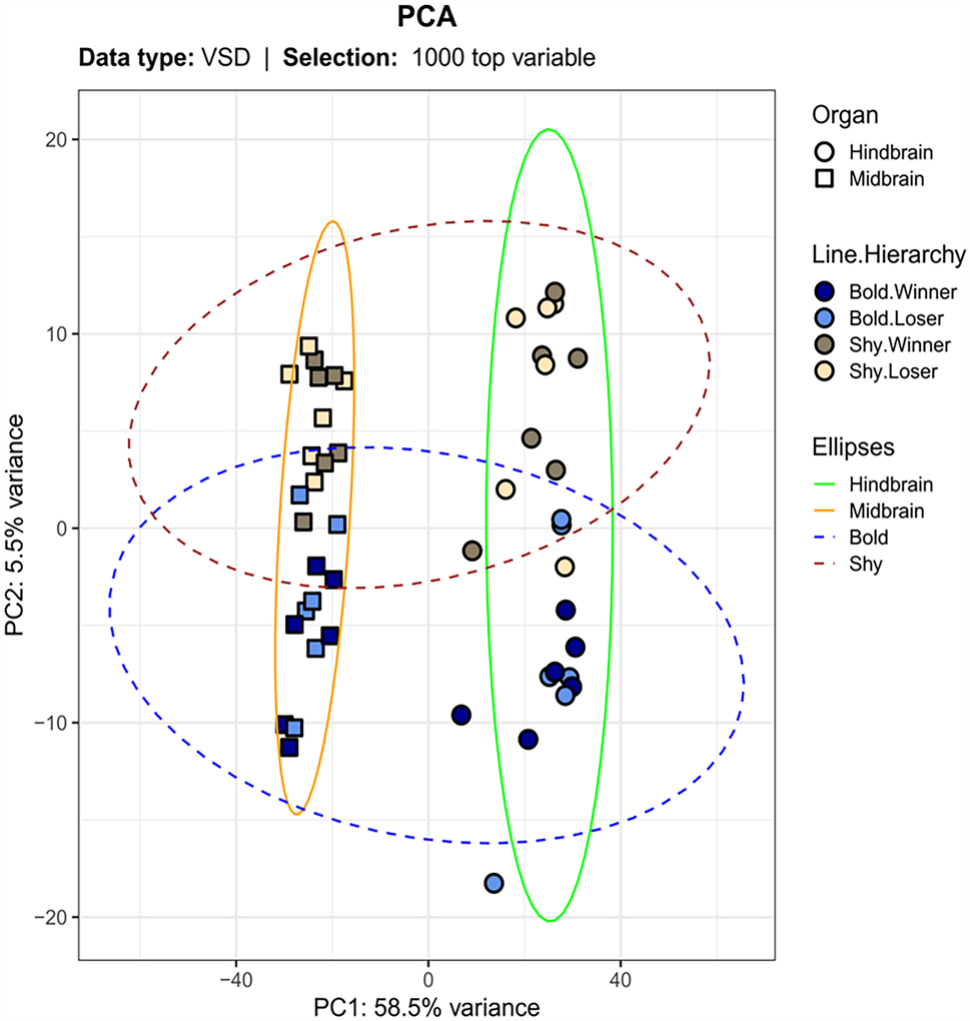
Principal Component Analysis (PCA) including the 1000 top variable genes in the Forebrain/Midbrain and Hindbrain of the groups defined by line (shy/bold) and social experience (loser/winner). Four clusters described by brain region and fish line are highlighted by ellipses. VSD, Variance Stabilizing Transformation. Twenty-four individuals were used for this analysis.

Principal Component Analysis (PCA) was used to evaluate the general differences in brain transcriptomic profiles between the selected lines and social experiences (Fig. 2). PC1 was explained by the difference between the sampled areas of the brain (FBMB, HB) at 58.5% variance. PC2 of the PCA was explained by the bold and shy stress coping style at 5.5% variance. No significant distinction between winners and losers could be estimated based on the PCA. Thus, the largest variability in the PCA analysis could be explained by the brain regions, followed by the stress-coping styles and social experience.

**Fig. 3:**
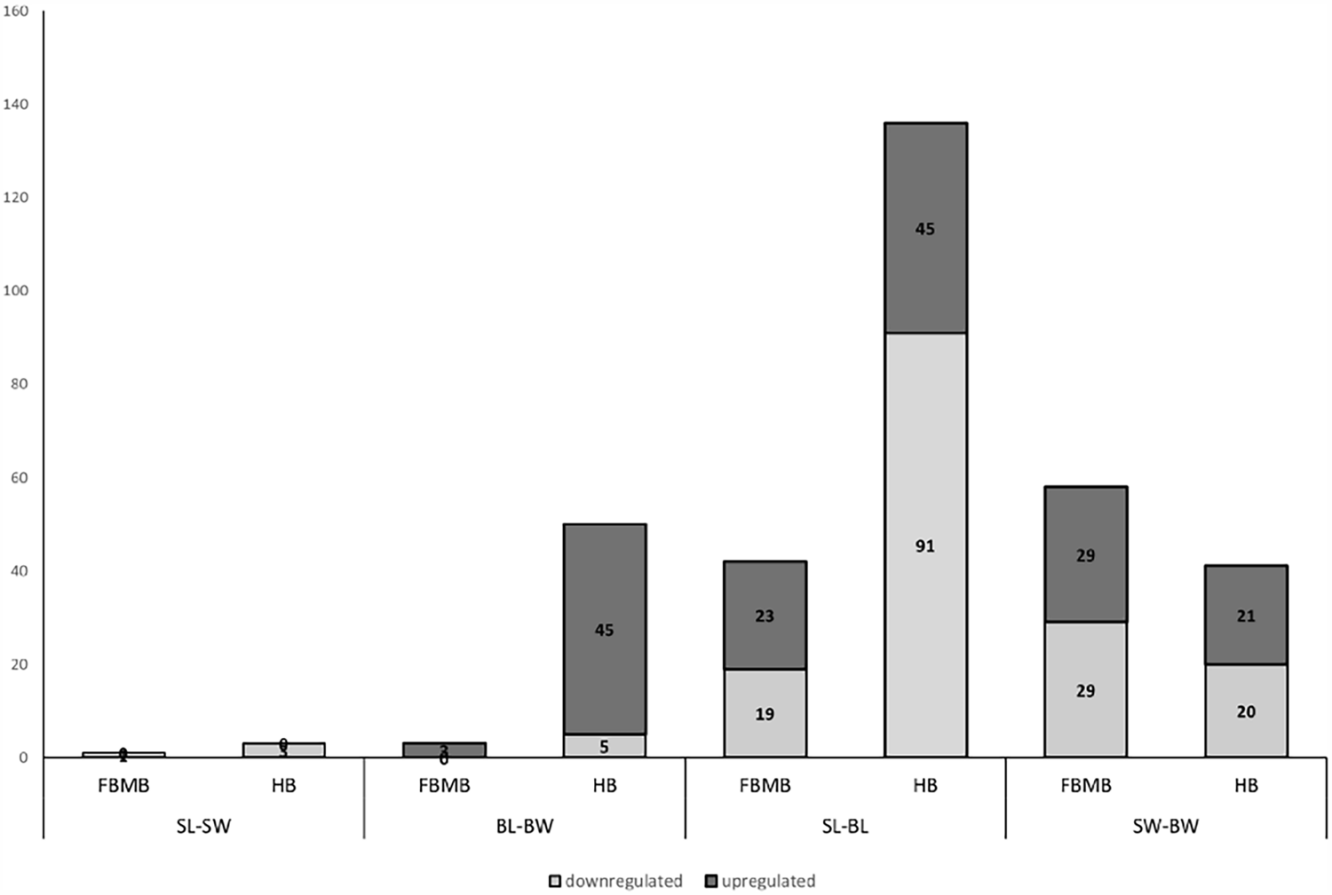
Numbers of differentially expressed genes in Forebrain & Midbrain (FBMB) and Hindbrain (HB) of groups with divergent behavioural phenotypes and social experience. SL-SW, Shy Loser *vs* Shy Winner; BL-BW, Bold Loser *vs* Bold Winner; SL-BL, Shy Loser *vs* Bold Loser; SW-BW, Shy Winner *vs* Bold Winner. Twenty-four individuals were used for this analysis.

**Fig. 4:**
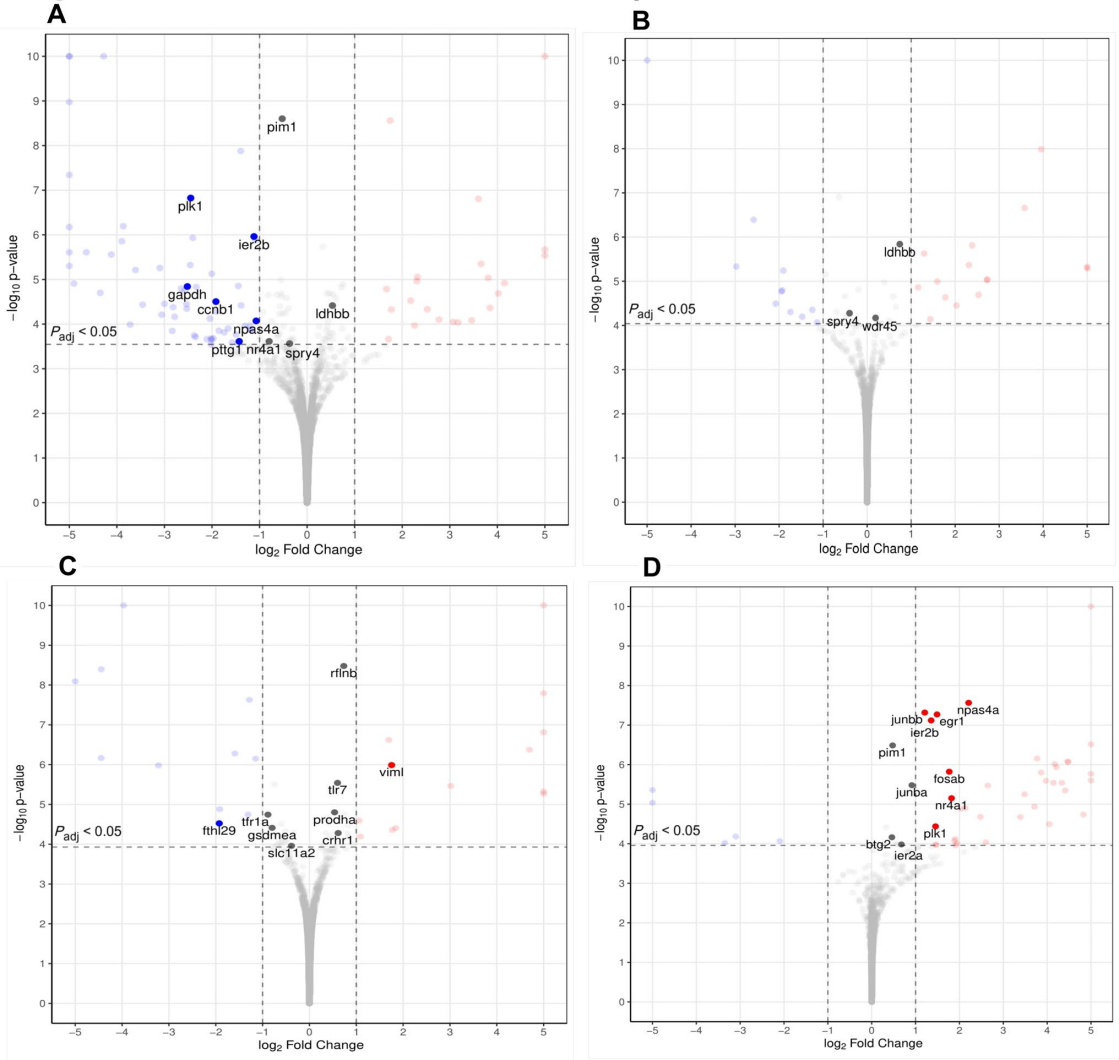
Volcano plots representing the differentially expressed genes in **A.** HB shy losers *vs* bold losers; **B** FBMB shy losers vs bold losers; **C** MB shy winners vs bold winners; **D** FBMB bold losers vs bold winners. Red dots represent upregulated transcripts, and blue dots represent downregulated transcripts in the given group (*P*-value ≤ 0.05 and log2 Fold Change > 1). Twenty-four individuals were used for this analysis.

The differences in the PCA were reflected in the number of differentially expressed genes (DEGs) (Fig. 3). There were marked differences based on the selected line within particular brain regions (see Supplementary File SF-3). In the hindbrain, the shy losers had downregulated the immediate early response genes (*ier2b*), neuronal PAS domain protein 4a (*npas4a*), and nuclear receptor subfamily 4, group A, member 1 (*nr4a1*) involved in neuronal development and plasticity in comparison to bold losers. In addition, several genes linked to possible oncogenesis (*ccnb1, plk1, pim1, pttg1, spry4*) or senescence (*pim1, gapdh, ldhbb*) were downregulated, except for the *ldhbb* gene, which was upregulated (Fig. 4A). In forebrain/midbrain, the losers of the shy *vs* bold line manifested differences in a particular gene: WD repeat domain 45 (*wdr45*) was upregulated in shy losers compared to bold losers (Fig. 4B). The shy winners showed upregulation in the forebrain/midbrain region in genes contributing to neuronal development, plasticity, and memory formation, such as proline dehydrogenase 1a (*prodha*), toll-like receptor 7 (*tlr7*), and vimentin-like *(viml*) compared to bold winners. The corticotropin-releasing hormone receptor 1 (*crhr1*) involved in the HPI axis of fish was upregulated in shy winners compared to bold. DEGs of other significant processes in the forebrain/midbrain were downregulated in shy winners compared to bold: genes involved in iron metabolism (*fthl29, slc11a2, tfr1a)*, and pyroptosis (*gsdmea, rflnb*). All were downregulated in shy *vs* bold winners, apart from the *rflnb* gene (Fig. 4C). Despite the significant change in gene expression, some of the genes (*nr4a1, spry4, ldhbb, pim1, wdr45, tlr7, rflnb, prodha, crhr1, gsdmea, slc11a2*) did not reach the pre-defined cut-off of 2-fold change.

The bold and shy fish responded differently to social challenges. There were few transcriptomic changes between winners and the losers in the shy line in any of the brain regions. Specifically, the shy fish differed very little in their transcriptomic profiles, which was intriguing. Bold fish, however, responded significantly to social experiences. The socially regulated genes in the hindbrain in the bold line (loser *vs* winner) included upregulation of B-cell Translocation gene 2 (*btg2*), an early growth response gene (*egr1*), FBJ osteosarcoma oncogene (*fosab*), immediate early response genes (*ier2a, ier2b*), jun B proto-oncogenes (*junba, junbb*), neuronal PAS domain protein 4a (*npas4a*), and nuclear receptor subfamily 4, group A, member 1 (*nr4a1*), which are involved in neuronal development and plasticity (*btg2, egr1, junba, junbb, npas4a, nr4a1*). Specific genes, such as *pim1* and *plk1,* were upregulated, which are potentially linked to oncogenesis (Fig. 4D). The pre-defined cutoff value of 2-fold change was not reached by *pim1, junba, btg2*, and *ier2a*, even though their expression was significantly altered. In the forebrain/midbrain region, only a single gene was differentially expressed between shy winners and losers, and only 3 DEGs were detected between the bold winners and losers, which precludes solid conclusions based on these changes. Combined, the effects of social experience were most pronounced in bold fish and involved genes associated with neural development, plasticity, and potentially oncogenesis.

## Discussion

In this study, we demonstrated that zebrafish selected for distinct behavioural phenotypes exhibit differing responses to social interactions. Furthermore, these variations in behaviour are associated with differences in the transcriptomic profiles of the forebrain/midbrain and hindbrain in bold and shy fish. However, our findings indicate that in zebrafish, the intrinsic behavioural profile, or stress coping style, is a more significant influence on behaviour and brain gene expression than the experience of social interaction.

### Stress Coping Styles

Our results demonstrate significant behavioural and transcriptomic differences between male zebrafish selected for contrasting boldness phenotypes. Fish selected for boldness or shyness were tested first for aggression and displacement behaviour, which allows a clear distinction between bold and shy fish without considering social rank (winner and loser). Consequently, the other behavioural parameters were tested after repeated social experience, and, hence, the social experience (winner, loser) was considered in the analysis. In other words, the stress coping styles are assessed as differences between bold and shy winners as well as between bold and shy losers. The bold individuals exhibited increased aggression (3 significant differences) prior to any induced social interactions. They also demonstrated significantly enhanced boldness and exploratory behaviours (16 significant differences), along with increased locomotor activity in the zMCSF arena (10 significant differences). The variation in risk-taking behaviour or boldness has been shown to differ significantly among proactive and reactive individuals in early developmental stages of zebrafish, flathead grey mullet (*Mugil cephalus*), common carp (*Cyprinus carpio*), and sea bream [33, 46–49] The selection programme in this study produced significant differences within the population, which behaviourally align with the distinctions between proactive and reactive stress coping styles [11]. These differences were distinct despite varying social experiences, demonstrating consistency over time and across different situations [1].

The selected lines are anticipated to exhibit variations in stress responses, which will subsequently be apparent in their transcriptomic profiles. A key characteristic of stress coping styles is the divergence in reactivity of the HPI axis [10]. In the present study, bold winners had a significantly lower expression of the *crh1* receptor gene than shy winners, indicating a reduced responsiveness to stress *via* the HPI axis in this cohort. Following activation of the HPI axis, the stress hormone cortisol is released [50, 51]. Notably, bold losers were indistinguishable from shy losers regarding the expression of stress markers, suggesting that recent social experiences had enduring effects on the reactivity of the HPI axis, thereby diminishing selected line differences. It has been suggested that low social rank may lead to chronic stress, as these individuals typically display behavioural inhibition and submissive postures [52, 53]. The expression of the *crh1* receptor gene could have been increased in bold losers, likely as a result of the chronic stress they encountered, given that the *crh1* receptor gene is associated with anxiety [54].

**Fig. 5:**
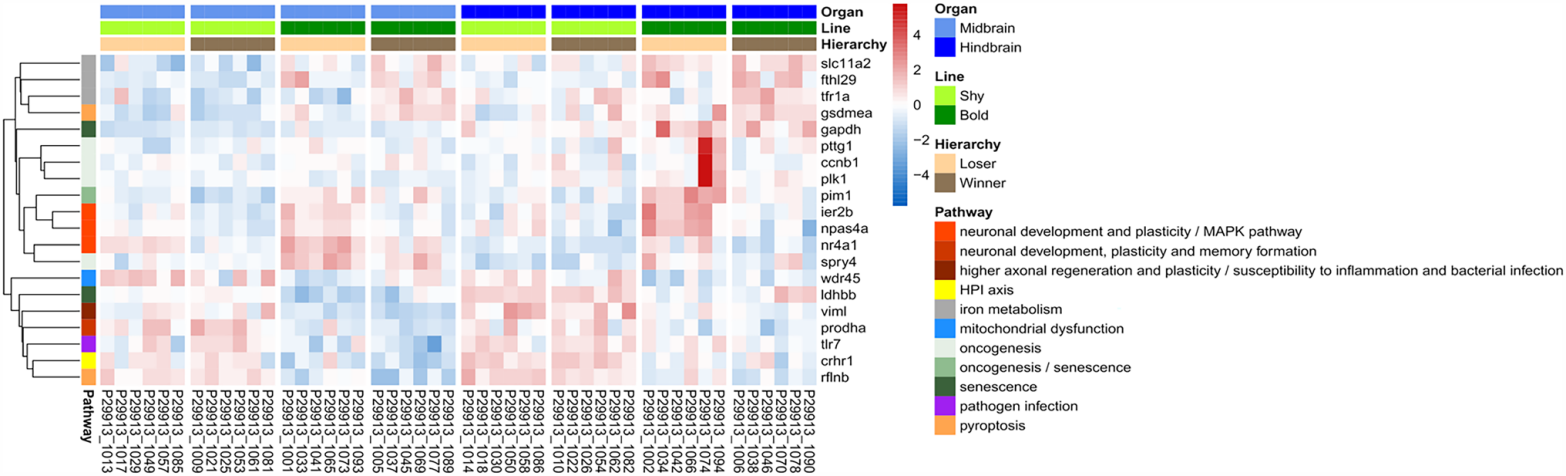
Graphical representation of biological processes and pathways associated with differentially expressed genes in the midbrain/forebrain and hindbrain of groups from different lines and social experience. The pathways were collected from a literature search and based on the STRING database (https://string-db.org/).

The transcriptomic profiles of shy and bold fish were influenced by their social experiences. Bold *vs* shy losers had upregulated the immediate early genes involved in the mitogen activated protein kinase (MAPK) pathway (*ier2b, npas4a, nr4a1*; WikiPathways; STRING database; Fig. 5). These genes function as transcription factors, regulating essential cellular processes such as the cell cycle and the balance between excitatory and inhibitory signals [55–57]. Chronic social defeat has been shown to elevate the expression of these genes, as reported in studies involving mice [58]. Typically, these genes are associated with neural plasticity [58–60]. Shy losers exhibited an upregulation of the *wdr45* gene in the forebrain/midbrain, which is known to enhance cognitive function [61]. Even though the stress did not induce as prominent an activation of the MAPK pathway in shy losers that would support the neural plasticity proposition, they had a predisposition for learning ability due to the *wdr45* gene in the forebrain/midbrain region.

The bold losers exhibited notable differences from shy losers in several oncogenic markers, including *ccnb1, plk1, pim1, pttg1,* and *spry4*, which were found to be upregulated. The STRING database indicated that the *plk1, pttg1*, and *ccnb1* genes are co-expressed and connected to the cell cycle pathways in KEGG and WikiPathways. Current literature suggests that these markers are more closely associated with the growth of microglia and astrocytes rather than neuronal proliferation [62–65]. Consequently, bold losers are at a significantly higher risk for microglial cell proliferation compared to their shy counterparts, which demonstrate the expression of several genes linked to cellular arrest, apoptosis, and senescence, such as *pim1, gpdh,* and *ldhbb* [66].

The analysis of the transcriptomic profiles of bold and shy winners revealed significant differences, suggesting distinct predispositions compared to those between bold and shy losers. In terms of neural plasticity, shy winners exhibited upregulation of the vimentin-like gene (*viml*). This gene's corresponding protein serves a dual role; while it stabilises the intracellular structure of intermediate filaments and enhances axonal regeneration and plasticity, it concurrently increases the risk of susceptibility to inflammation and bacterial infections [67, 68]. Shy winners, therefore, appear to be predisposed to greater synaptic plasticity, but also potentially more vulnerable to pathogen infections. This is further evidenced by the overexpression of the toll-like receptor 7 (*tlr7*) gene, which has been associated with heightened levels in tissues affected by pathogen infections [69]. One of the primary mechanisms for pathogen elimination is pyroptosis, a form of cell death initiated by inflammasomes [70]. The gene expression from the current study indicates that shy winners are indeed at a higher risk of pathogen infection, as demonstrated by the expression levels of genes associated with pyroptosis (upregulation of *rflnb* and downregulation of *gdsmea*) [71], which could also lead to neuroinflammation. Prior research has shown that chronic elevation of stress hormones is correlated with increased mortality from common fungal and bacterial diseases [72]. More recently, reactive fish, classified based on post-stress cortisol levels and skin pigmentation, have been shown to be more susceptible to infection by sea lice (*Lepeophtheirus salmonis*) [73]. Therefore, shy winners are likely to be at a greater risk for infections and infection-related diseases.

On the contrary, bold winners exhibited signs of iron accumulation in their brains, as indicated by upregulation of the ferritin (*fthl29*), transferrin receptor (*tfr1a*), and *slc11a2* genes. These genes are implicated in ferroptosis within the KEGG pathways, iron ion transmembrane transporter activity (local network cluster), and abnormal nucleate erythrocyte low saturation (Monarch; STRING database). Transferrin facilitates the endocytosis of iron ions across the blood-brain barrier, allowing for their transport to cells [74]. Excessive free iron ions can lead to oxidative damage in cells due to their role in promoting reactive oxygen species (ROS) production [75]. Ferritin is upregulated in response to increased intracellular iron ion concentrations, serving to store iron in an inactive form [75, 76]. The *slc11a2* gene is associated with iron overload in the brain, although its activity leads to the release and export of stored iron [77]. The upregulation of this gene suggests that bold winners release more iron from storage than their shyer counterparts. The upregulation of genes involved in iron metabolism and the consequent iron accumulation have been associated with cognitive impairment, major depressive disorder, type 2 diabetes, and neurodegenerative diseases in humans [68, 76, 78, 79]. Further research in this direction could improve the modelling of these diseases in zebrafish if replication further supports these findings.

### Repeated Social Experience

The shy/reactive and bold/proactive coping styles responded differently to social challenge. In bold individuals, the primary difference between losers and winners was in the latency to enter the CORN zone, associated with diminished exploratory behaviour in rodents [80]. A similar increase in latency to enter the corner in the MCSF arena has been found in rats bred for high alcohol uptake [80] and in shelter-seeking rats [81]; however, these behavioural changes were accompanied by additional modifications not seen in the current study. Consequently, the social challenge exerted only weak effects on the activity of bold individuals in this investigation. Conversely, substantial alterations in transcriptomics were evident between bold winners and losers. Nine DEGs, namely *ier2a, ier2b, junba, junbb, fosab, btg2, egr1, nr4a1,* and *npas4a*, showed co-expression in the STRING database and are implicated in significant biological pathways. These DEGs are associated with the regulation of transcription and DNA-binding activity in the nucleus, as defined by Gene Ontology classifications for both Molecular Function and Cellular Component (STRING database). The fibroblast growth factor (FGF) signalling pathway involves the *junba, fosab* and *egr1* genes (WikiPathways; STRING) and is regulated by the expression of the *ier2* gene [82]. This signalling pathway is crucial for neuronal growth and differentiation, and it recruits adapter proteins from the ERK1/ERK2 MAPK pathway [83]. The Jun proteins and Fosab are included among these recruited adapter proteins, functioning downstream of the FGF signalling pathway. Consequently, FGFs may inhibit neuronal cell death and potentially promote oncogenesis if excessively activated [84], while also facilitating improved spatial learning and neurogenesis [83]. FGFs are essential in the crosstalk between neurons, microglia, and astrocytes [85]. The activity of astrocytes is tightly regulated due to the risk of chronic inflammation, with FGFs helping to maintain their nonreactive state [85]. The upregulation of the *pim1* and *plk1* genes in bold losers in the current study supports the possibility of tumour formation or its predisposition. Upregulation of similar genes that were found in the present study (*npas4a, fosab, nr4a1, ier2*) was found in losers compared to winners in zebrafish [86]. These genes are believed to relate to the fish's self-assessment of its social rank [86], which aligns with the findings of the present study. Consequently, the bold fish demonstrated a response to their social experience by modifying their brain transcriptome, yet this did not translate into a change in behaviour.

In contrast, shy or reactive fish did not exhibit significant changes in their brain transcriptomes, although their behaviour underwent notable alterations. Specifically, the shy losers manifested an increase in the latency to attack, swam more rapidly through the CIRC zone, and had higher locomotor activity as evidenced by their movement through the corridors in the zMCSF arena [37]. These behavioural differences in shy losers may indicate elevated anxiety, which manifests as more intense burst swimming and erratic movements [87–89]. Anxiety responses can either involve behavioural disinhibition, such as increased locomotor activity, or inhibition, like freezing [90]. The preference for one type of anxiety response is recognized as a crucial factor in determining stress coping styles [90]. The freezing response is typically the natural reaction to stress in shy or reactive fish [90], contrasting with the behaviour observed in this study. In addition, startled fish tend to take longer to approach an opponent and initiate a fight, and when they do engage, they show less aggressive behaviours [91]. The response of the shy losers seems to resemble the response of startled fish. Conversely, shy winners markedly increased their aggressive actions, indicating a winner effect [18]. However, only minimal differences in the brain transcriptomes were found between shy winners and losers. This suggests a potential link between the anticipated increased plasticity of shy fish [92] and their ability to mitigate the impact of social defeat on the brain transcriptome by altering their behaviour. Behavioural flexibility empowers individuals to modify their actions in response to environmental stimuli [1]. Thus, the capacity of shy losers to reduce the negative effects of chronic stress from dyadic encounters on their brain transcriptomes may stem from their ability to adjust their behaviour, highlighting a greater level of behavioural flexibility in shy fish within social contexts.

### Limitations of the Study

It was not feasible to prevent the occurrence of potential siblings in the current study. The mirror test is a widely utilized non-invasive method for assessing aggression in fish [93]; however, it is important to recognize that engaging with a mirror image may differ significantly from interacting with a live opponent [94]. Transcriptomic analysis focused on large brain regions (forebrain/midbrain and hindbrain), which limited the ability to conduct detailed examinations of specific nuclei. A selection of more defined brain areas may yield a clearer and more comprehensive understanding of our findings.

## Supplementary Information

Below is the link to the electronic supplementary material.

Supplementary file 1 (PDF 1061 KB)

## Data Availability

The data reported in this paper will be shared by the lead contact upon request. Any additional information required to reanalyse the data reported in this paper is available from the lead contact upon request.

## Generative AI and AI-Assisted Technologies

During the preparation of this work, we used AvidNote in order to improve the readability of the text. After using this tool/service, we reviewed and edited the content as needed and take(s) full responsibility for the content of the published article.
