# Supplementary material for: Effects of Stress Coping Styles and Social Defeat on Zebrafish Behaviour and Brain Transcriptomics
Source: Neurosci Bull. 2025 Sep 22;42(5):989–1003. doi: 10.1007/s12264-025-01506-0 (PMC13158335; doi:10.1007/s12264-025-01506-0)
Supplement: Supplementary file 1 — (PDF 1061 KB) [file 12264_2025_1506_MOESM1_ESM.pdf]

Supplementary Files

SF-1 Artificial Selection

Selective breeding for boldness was used to generate lines of zebrafish manifesting a divergent behavioural phenotype. The founder population were wild-caught zebrafish captured in West Bengal, India. Their offspring (n = 1000) spawned on site were transported to Norwegian Technical University (Trondheim, Norway). Eggs collected from the offspring of wild-caught zebrafish, the F_0 generation, were transported to Uppsala University (Uppsala, Sweden; courtesy of Dr. Fredrik Jutfelt). The adult fish (F_0) were tagged by p-Chip (0.5 mm × 0.5 mm × 0.1 mm) according to the p-Chip Implantation Protocol (Chen *et al.*, 2017), except for the fact that 200 mg/L of benzocaine (Merck KGaA, Darmstadt, Germany) was used for anaesthesia in the present study. The fish were consequently tested twice in the novel tank diving (NTD) test for boldness (latency to enter the top zone of the tank) according to Thörnqvist *et al.* (2019). The fish (250-600 individuals) were selected from the upper and lower extremes of the population, i.e., the fish being boldest and the fish being the shyest. Proactive (bold) and reactive (shy) lines were generated in duplicate (i.e., R1 shy, R2 shy, R1 bold, R2 bold) and randomly mated within the same selected line. Parents (125-300 fish) were selected for mating of the F_1 generation. The same process was repeated to generate the F_2 generation. The F_2 generation of fish significantly differed in boldness in the NTD test (see **SF-1**).

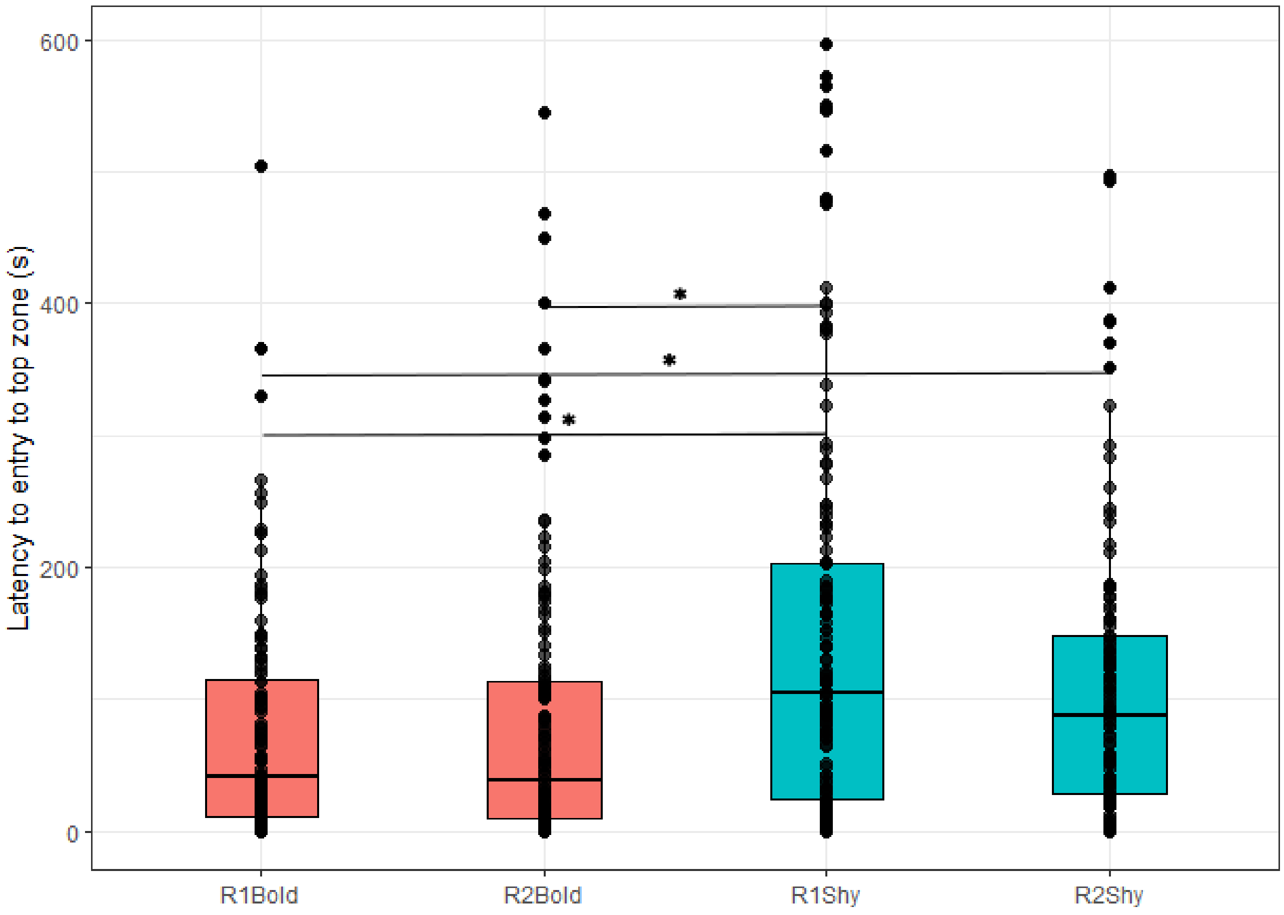

SF-1: The differences in the latency to enter the top zone between bold and shy selected lines generated in duplicate (R1, R2 lines for both shy and bold fish) of the F_2 generation of fish. Ordernorm transformation was used to normalise the data. Linear mixed effect models with Line as a fixed effect and individual as a random effect were applied to detect significant differences at $\alpha = 0.05$ and are highlighted with an asterisk. The emmeans function was as a pairwise comparison between selected lines.

SF-2: Results of the contrast analysis using the "emmeans" package in R from the Mirror Test (MT) and the zebrafish Multivariate Concentric Square Field (zMCSF) test. The non-normal data were transformed based on the recommendation of the "bestNormalize" package in R. 1 = the first trial of MT, 1-2 = the percentage change from the first to the second trial of MT, B = bold line, BL = bold loser, BW = bold winner, NB = negative binomial distribution, oN = orderNorm transformation, PD = Poisson distribution; S = shy line, SL = shy loser, sqrt = square-root transformation, SW = shy winner, YJ = Yeo Johnson transformation.

Comparisons	Test	df	Test statistics value	p_{adj}
**MT – Number of aggressive interactions**				
B (1) – S (1)	GLMM (PD)		2.78	.005**
BL (1-2) – BW (1-2)	ANOVA (oN)	28	1.44	.160
SL (1-2) – SW (1-2)	ANOVA (oN)	28	-1.03	.0312
BL (1-2) – SL (1-2)	ANOVA (oN)	28	-0.16	.875
BW (1-2) – SW (1-2)	ANOVA (oN)	28	-2.63	.014*
**MT – Latency to the first attack**				
B (1) – S (1)	ANOVA (log)	30	0.65	.520
BL (1-2) – BW (1-2)	ANOVA (oN)	28	-0.92	.365
SL (1-2) – SW (1-2)	ANOVA (oN)	28	1.38	.180
BL (1-2) – SL (1-2)	ANOVA (oN)	28	-2.28	.031*
BW (1-2) – SW (1-2)	ANOVA (oN)	28	0.02	.985
**MT – Total duration of attack**				
B (1) – S (1)	ANOVA (oN)	30	2.71	.011*
BL (1-2) – BW (1-2)	ANOVA (oN)	28	0.08	.934
SL (1-2) – SW (1-2)	ANOVA (oN)	28	-0.64	.529
BL (1-2) – SL (1-2)	ANOVA (oN)	28	0.18	.860
BW (1-2) – SW (1-2)	ANOVA (oN)	28	-0.54	.592
**MT – Average duration of attack**				
B (1) – S (1)	ANOVA (oN)	30	2.45	.020*
BL (1-2) – BW (1-2)	ANOVA (oN)	28	-0.84	.411
SL (1-2) – SW (1-2)	ANOVA (oN)	28	0.04	.970
BL (1-2) – SL (1-2)	ANOVA (oN)	28	-0.50	.624
BW (1-2) – SW (1-2)	ANOVA (oN)	28	0.38	.709
**MT – Number of displaced behaviours**				
B (1) – S (1)	GLMM (NB)		-0.40	.690
BL (1-2) – BW (1-2)	ANOVA (oN)	28	0.55	.584
SL (1-2) – SW (1-2)	ANOVA (oN)	28	1.56	.131
BL (1-2) – SL (1-2)	ANOVA (oN)	28	-0.33	.745
BW (1-2) – SW (1-2)	ANOVA (oN)	28	0.67	.507
**MT – Latency to the first displaced behaviour**				
B (1) – S (1)	ANOVA (oN)	30	-0.87	.391
BL (1-2) – BW (1-2)	ANOVA (oN)	28	0.04	.981
SL (1-2) – SW (1-2)	ANOVA (oN)	28	-0.48	.637
BL (1-2) – SL (1-2)	ANOVA (oN)	28	0.42	.676
BW (1-2) – SW (1-2)	ANOVA (oN)	28	-0.08	.938
**MT – Total duration of displaced behaviour**				
B (1) – S (1)	ANOVA (oN)	30	-0.49	.631
BL (1-2) – BW (1-2)	ANOVA (oN)	28	-0.19	.853
SL (1-2) – SW (1-2)	ANOVA (oN)	28	1.15	.260
BL (1-2) – SL (1-2)	ANOVA (oN)	28	-0.14	.892
BW (1-2) – SW (1-2)	ANOVA (oN)	28	1.20	.241
**MT – Average duration of displaced behaviour**				
B (1) – S (1)	ANOVA (oN)	30	-0.65	.523
BL (1-2) – BW (1-2)	ANOVA (oN)	28	-0.69	.496
SL (1-2) – SW (1-2)	ANOVA (oN)	28	0.52	.610
BL (1-2) – SL (1-2)	ANOVA (oN)	28	0.33	.747
BW (1-2) – SW (1-2)	ANOVA (oN)	28	1.53	.137
**zMCSF – Total distance moved (arena)**				
BL - BW	ANOVA	28	-0.18	.861
BL - SL	ANOVA	28	1.16	.256
BW - SW	ANOVA	28	0.99	.333
SL - SW	ANOVA	28	-0.35	.728
**zMCSF – Average velocity (arena)**				
BL - BW	ANOVA	28	-0.25	.808
BL - SL	ANOVA	28	0.99	.332
BW - SW	ANOVA	28	1.01	.323
SL - SW	ANOVA	28	-0.23	.822

**zMCSF – Total activity (arena)**				
BL - BW	ANOVA	28	-0.28	.783
BL - SL	ANOVA	28	1.13	.269
BW - SW	ANOVA	28	1.15	.260
SL - SW	ANOVA	28	-0.26	.800
**zMCSF – Immobility (arena)**				
BL - BW	ANOVA (sqrt)	28	-0.37	.715
BL - SL	ANOVA (sqrt)	28	-1.00	.326
BW - SW	ANOVA (sqrt)	28	0.48	.638
SL - SW	ANOVA (sqrt)	28	1.11	.278
**zMCSF – Total distance moved (zones)**				
START: BL – BW	LMM (YJ)	180	0.32	.751
START: BL - SL	LMM (YJ)	180	0.84	.401
START: BW - SW	LMM (YJ)	171	0.29	.773
START: SL – SW	LMM (YJ)	171	-0.25	.802
DCR: BL – BW	LMM (YJ)	180	0.21	.832
DCR: BL - SL	LMM (YJ)	180	-0.31	.759
DCR: BW - SW	LMM (YJ)	171	-0.84	.403
DCR: SL – SW	LMM (YJ)	171	-0.30	.762
CORR1: BL – BW	LMM (YJ)	171	-0.66	.507
CORR1: BL - SL	LMM (YJ)	195	0.14	.888
CORR1: BW - SW	LMM (YJ)	171	1.56	.120
CORR1: SL – SW	LMM (YJ)	195	0.70	.484
CORN: BL – BW	LMM (YJ)	180	-0.92	.359
CORN: BL - SL	LMM (YJ)	218	-0.20	.845
CORN: BW - SW	LMM (YJ)	171	2.15	.033*
CORN: SL – SW	LMM (YJ)	211	1.28	.203
CORR2: BL – BW	LMM (YJ)	180	-1.02	.312
CORR2: BL - SL	LMM (YJ)	202	0.01	.993
CORR2: BW - SW	LMM (YJ)	171	2.53	.012*
CORR2: SL – SW	LMM (YJ)	195	1.39	.166
RAMP1: BL – BW	LMM (YJ)	180	0.31	.759
RAMP1: BL - SL	LMM (YJ)	202	0.71	.478
RAMP1: BW - SW	LMM (YJ)	171	1.56	.122
RAMP1: SL - SW	LMM (YJ)	195	1.03	.304
RAMP2: BL – BW	LMM (YJ)	180	0.53	.596
RAMP2: BL - SL	LMM (YJ)	190	2.45	.015*
RAMP2: BW - SW	LMM (YJ)	171	0.20	.841
RAMP2: SL – SW	LMM (YJ)	182	-1.79	.075
RAMP3: BL – BW	LMM (YJ)	180	0.13	.898
RAMP3: BL - SL	LMM (YJ)	180	2.58	.011*
RAMP3: BW - SW	LMM (YJ)	171	0.89	.374
RAMP3: SL – SW	LMM (YJ)	171	-1.63	.105
RAMP4: BL – BW	LMM (YJ)	190	-1.58	.116
RAMP4: BL - SL	LMM (YJ)	180	1.68	.094
RAMP4: BW - SW	LMM (YJ)	182	3.59	< .001***
RAMP4: SL - SW	LMM (YJ)	171	0.29	.771
CIRC: BL – BW	LMM (YJ)	203	0.20	.845
CIRC: BL – SL	LMM (YJ)	190	0.55	.586
CIRC: BW – SW	LMM (YJ)	204	0.01	.996
CIRC: SL – SW	LMM (YJ)	192	-0.34	.735
CENT: BL – BW	LMM (YJ)	171	0.62	.539
CENT: BL – SL	LMM (YJ)	171	1.57	.119
CENT: BW – SW	LMM (YJ)	171	-0.49	.623
CENT: SL – SW	LMM (YJ)	171	-1.44	.151
REST: BL – BW	LMM (YJ)	171	-0.03	.976
REST: BL – SL	LMM (YJ)	171	0.07	.946
REST: BW - SW	LMM (YJ)	171	0.18	.856
REST: SL - SW	LMM (YJ)	171	0.09	.932
**zMCSF – Average velocity (zones)**				

START: BL – BW	LMM (oN)	119	-0.88	.382
START: BL - SL	LMM (oN)	119	1.18	.239
START: BW - SW	LMM (oN)	111	1.54	.126
START: SL – SW	LMM (oN)	111	-0.57	.570
DCR: BL – BW	LMM (oN)	119	-0.07	.947
DCR: BL - SL	LMM (oN)	119	0.69	.491
DCR: BW - SW	LMM (oN)	111	0.44	.659
DCR: SL – SW	LMM (oN)	111	-0.34	.738
CORR1: BL – BW	LMM (oN)	111	0.26	.795
CORR1: BL - SL	LMM (oN)	129	0.57	.568
CORR1: BW - SW	LMM (oN)	111	-0.14	.888
CORR1: SL – SW	LMM (oN)	129	-0.46	.647
CORN: BL – BW	LMM (oN)	119	-0.15	.880
CORN: BL - SL	LMM (oN)	150	-0.50	.621
CORN: BW - SW	LMM (oN)	111	-1.21	.230
CORN: SL – SW	LMM (oN)	143	-0.74	.459
CORR2: BL – BW	LMM (oN)	119	-0.27	.784
CORR2: BL - SL	LMM (oN)	136	0.21	.837
CORR2: BW - SW	LMM (oN)	111	0.24	.808
CORR2: SL – SW	LMM (oN)	129	-0.25	.806
RAMP1: BL – BW	LMM (oN)	119	-0.09	.932
RAMP1: BL - SL	LMM (oN)	136	0.20	.845
RAMP1: BW - SW	LMM (oN)	111	0.76	.452
RAMP1: SL - SW	LMM (oN)	129	0.43	.666
RAMP2: BL – BW	LMM (oN)	119	0.23	.817
RAMP2: BL - SL	LMM (oN)	126	1.64	.104
RAMP2: BW - SW	LMM (oN)	111	0.65	.515
RAMP2: SL – SW	LMM (oN)	119	-0.81	.421
RAMP3: BL – BW	LMM (oN)	119	-0.80	.427
RAMP3: BL - SL	LMM (oN)	119	0.65	.515
RAMP3: BW - SW	LMM (oN)	111	1.03	.304
RAMP3: SL – SW	LMM (oN)	111	-0.46	.649
RAMP4: BL – BW	LMM (oN)	126	-0.05	.962
RAMP4: BL - SL	LMM (oN)	119	-0.20	.839
RAMP4: BW - SW	LMM (oN)	119	0.61	.540
RAMP4: SL - SW	LMM (oN)	111	0.79	.433
CIRC: BL – BW	LMM (oN)	136	0.85	.398
CIRC: BL – SL	LMM (oN)	126	-0.09	.931
CIRC: BW – SW	LMM (oN)	136	1.02	.312
CIRC: SL – SW	LMM (oN)	126	2.00	.048*
CENT: BL – BW	LMM (oN)	111	-0.37	.713
CENT: BL – SL	LMM (oN)	111	1.97	.052
CENT: BW – SW	LMM (oN)	111	2.49	.014*
CENT: SL – SW	LMM (oN)	111	0.16	.876
REST: BL – BW	LMM (oN)	111	-0.36	.721
REST: BL – SL	LMM (oN)	111	-0.59	.559
REST: BW - SW	LMM (oN)	111	1.45	.150
REST: SL - SW	LMM (oN)	111	1.68	.096
**zMCSF – Total duration in zone (zones)**				
START: BL – BW	LMM (logit)	265	0.52	.603
START: BL - SL	LMM (logit)	265	-0.00	.997
START: BW - SW	LMM (logit)	260	-0.10	.921
START: SL – SW	LMM (logit)	260	0.44	.659
DCR: BL – BW	LMM (logit)	265	-0.12	.904
DCR: BL - SL	LMM (logit)	265	-1.15	.252
DCR: BW - SW	LMM (logit)	260	-0.71	.481
DCR: SL – SW	LMM (logit)	260	0.36	.721
CORR1: BL – BW	LMM (logit)	260	0.55	.581
CORR1: BL - SL	LMM (logit)	275	0.77	.444
CORR1: BW - SW	LMM (logit)	260	1.38	.169

CORR1: SL – SW	LMM (logit)	275	1.03	.304
CORN: BL – BW	LMM (logit)	265	-1.06	.292
CORN: BL - SL	LMM (logit)	286	0.01	.993
CORN: BW - SW	LMM (logit)	260	2.65	.009**
CORN: SL – SW	LMM (logit)	284	1.37	.172
CORR2: BL – BW	LMM (logit)	265	-0.98	.327
CORR2: BL - SL	LMM (logit)	278	0.52	.602
CORR2: BW - SW	LMM (logit)	260	2.27	.024*
CORR2: SL – SW	LMM (logit)	275	0.63	.528
RAMP1: BL – BW	LMM (logit)	265	0.36	.721
RAMP1: BL - SL	LMM (logit)	278	0.76	.450
RAMP1: BW - SW	LMM (logit)	260	0.90	.369
RAMP1: SL - SW	LMM (logit)	275	0.40	.687
RAMP2: BL – BW	LMM (logit)	265	0.25	.806
RAMP2: BL - SL	LMM (logit)	271	2.36	.019*
RAMP2: BW - SW	LMM (logit)	260	0.29	.775
RAMP2: SL – SW	LMM (logit)	267	-1.91	.058
RAMP3: BL – BW	LMM (logit)	265	0.44	.659
RAMP3: BL - SL	LMM (logit)	265	2.35	.020*
RAMP3: BW - SW	LMM (logit)	260	0.61	.542
RAMP3: SL – SW	LMM (logit)	260	-1.36	.176
RAMP4: BL – BW	LMM (logit)	272	-1.25	.216
RAMP4: BL - SL	LMM (logit)	265	2.16	.032*
RAMP4: BW - SW	LMM (logit)	268	4.06	< .001***
RAMP4: SL - SW	LMM (logit)	260	0.63	.531
CIRC: BL – BW	LMM (logit)	279	0.31	.761
CIRC: BL – SL	LMM (logit)	271	0.78	.434
CIRC: BW – SW	LMM (logit)	281	-0.02	.984
CIRC: SL – SW	LMM (logit)	273	-0.49	.626
CENT: BL – BW	LMM (logit)	260	0.19	.853
CENT: BL – SL	LMM (logit)	260	0.60	.548
CENT: BW – SW	LMM (logit)	260	-1.13	.260
CENT: SL – SW	LMM (logit)	260	-1.55	.124
REST: BL – BW	LMM (logit)	260	-0.17	.867
REST: BL – SL	LMM (logit)	260	0.12	.909
REST: BW - SW	LMM (logit)	260	-0.22	.824
REST: SL - SW	LMM (logit)	260	-0.51	.614
**zMCSF – Duration of visit (zones)**				
START: BL – BW	LMM (logit)	247	0.28	.784
START: BL - SL	LMM (logit)	247	-1.95	.053
START: BW - SW	LMM (logit)	241	-0.49	.627
START: SL – SW	LMM (logit)	241	1.81	.072
DCR: BL – BW	LMM (logit)	247	-0.27	.789
DCR: BL - SL	LMM (logit)	247	-1.63	.104
DCR: BW - SW	LMM (logit)	241	-0.27	.785
DCR: SL – SW	LMM (logit)	241	1.14	.257
CORR1: BL – BW	LMM (logit)	241	1.26	.208
CORR1: BL - SL	LMM (logit)	259	0.00	.997
CORR1: BW - SW	LMM (logit)	241	0.69	.492
CORR1: SL – SW	LMM (logit)	259	1.82	.070
CORN: BL – BW	LMM (logit)	247	-0.38	.706
CORN: BL - SL	LMM (logit)	274	0.68	.499
CORN: BW - SW	LMM (logit)	241	1.74	.084
CORN: SL – SW	LMM (logit)	271	0.50	.616
CORR2: BL – BW	LMM (logit)	247	-0.78	.438
CORR2: BL - SL	LMM (logit)	264	0.66	.511
CORR2: BW - SW	LMM (logit)	241	0.44	.658
CORR2: SL – SW	LMM (logit)	259	-1.01	.312
RAMP1: BL – BW	LMM (logit)	247	0.13	.894
RAMP1: BL - SL	LMM (logit)	264	0.26	.796

RAMP1: BW - SW	LMM (logit)	241	0.32	.749
RAMP1: SL - SW	LMM (logit)	259	0.16	.871
RAMP2: BL – BW	LMM (logit)	247	-0.28	.778
RAMP2: BL - SL	LMM (logit)	255	0.66	.511
RAMP2: BW - SW	LMM (logit)	241	-0.13	.896
RAMP2: SL – SW	LMM (logit)	249	-1.09	.277
RAMP3: BL – BW	LMM (logit)	247	0.50	.616
RAMP3: BL - SL	LMM (logit)	247	0.80	.425
RAMP3: BW - SW	LMM (logit)	241	-0.54	.593
RAMP3: SL – SW	LMM (logit)	241	-0.84	.401
RAMP4: BL – BW	LMM (logit)	256	-0.60	.549
RAMP4: BL - SL	LMM (logit)	247	1.44	.152
RAMP4: BW - SW	LMM (logit)	250	2.63	.009**
RAMP4: SL - SW	LMM (logit)	241	0.60	.553
CIRC: BL – BW	LMM (logit)	265	0.33	.739
CIRC: BL – SL	LMM (logit)	255	0.28	.783
CIRC: BW – SW	LMM (logit)	267	-0.18	.862
CIRC: SL – SW	LMM (logit)	257	-0.11	.912
CENT: BL – BW	LMM (logit)	241	0.10	.921
CENT: BL – SL	LMM (logit)	241	-0.39	.697
CENT: BW – SW	LMM (logit)	241	-1.60	.111
CENT: SL – SW	LMM (logit)	241	-1.11	.267
REST: BL – BW	LMM (logit)	241	0.39	.694
REST: BL – SL	LMM (logit)	241	0.71	.476
REST: BW - SW	LMM (logit)	241	-0.79	.431
REST: SL - SW	LMM (logit)	241	-1.11	.268
**zMCSF – Number of visits to zone (zones)**				
START: BL – BW	GLMM (NB)		0.21	.830
START: BL - SL	GLMM (NB)		1.12	.263
START: BW - SW	GLMM (NB)		0.01	.989
START: SL – SW	GLMM (NB)		-0.92	.357
DCR: BL – BW	GLMM (NB)		0.05	.964
DCR: BL - SL	GLMM (NB)		-0.30	.761
DCR: BW - SW	GLMM (NB)		-0.55	.581
DCR: SL – SW	GLMM (NB)		-0.19	.852
CORR1: BL – BW	GLMM (NB)		-1.33	.184
CORR1: BL - SL	GLMM (NB)		-0.11	.917
CORR1: BW - SW	GLMM (NB)		1.34	.180
CORR1: SL – SW	GLMM (NB)		0.12	.906
CORN: BL – BW	GLMM (NB)		-0.78	.436
CORN: BL - SL	GLMM (NB)		-0.10	.924
CORN: BW - SW	GLMM (NB)		1.87	.061
CORN: SL – SW	GLMM (NB)		1.09	.277
CORR2: BL – BW	GLMM (NB)		-0.54	.590
CORR2: BL - SL	GLMM (NB)		-0.67	.502
CORR2: BW - SW	GLMM (NB)		2.33	.020*
CORR2: SL – SW	GLMM (NB)		2.37	.018*
RAMP1: BL – BW	GLMM (NB)		0.18	.855
RAMP1: BL - SL	GLMM (NB)		0.22	.824
RAMP1: BW - SW	GLMM (NB)		0.97	.333
RAMP1: SL - SW	GLMM (NB)		0.87	.385
RAMP2: BL – BW	GLMM (NB)		0.59	.553
RAMP2: BL - SL	GLMM (NB)		2.24	.025*
RAMP2: BW - SW	GLMM (NB)		0.14	.886
RAMP2: SL – SW	GLMM (NB)		-1.57	.117
RAMP3: BL – BW	GLMM (NB)		0.10	.922
RAMP3: BL - SL	GLMM (NB)		2.48	.013*
RAMP3: BW - SW	GLMM (NB)		0.95	.344
RAMP3: SL – SW	GLMM (NB)		-1.50	.133
RAMP4: BL – BW	GLMM (NB)		-1.27	.206

RAMP4: BL - SL	GLMM (NB)		2.18	.030*
RAMP4: BW - SW	GLMM (NB)		3.95	< .001***
RAMP4: SL - SW	GLMM (NB)		0.44	.657
CIRC: BL – BW	GLMM (NB)		0.40	.688
CIRC: BL – SL	GLMM (NB)		1.34	.179
CIRC: BW – SW	GLMM (NB)		-0.27	.786
CIRC: SL – SW	GLMM (NB)		-1.22	.224
CENT: BL – BW	GLMM (NB)		0.44	.660
CENT: BL – SL	GLMM (NB)		0.94	.349
CENT: BW – SW	GLMM (NB)		-0.52	.605
CENT: SL – SW	GLMM (NB)		-1.02	.309
REST: BL – BW	GLMM (NB)		-0.10	.923
REST: BL – SL	GLMM (NB)		-0.07	.942
REST: BW - SW	GLMM (NB)		0.20	.841
REST: SL - SW	GLMM (NB)		0.18	.860
**zMCSF – Number of visits (%) (zones)**				
START: BL – BW	LMM (logit)	306	0.33	.742
START: BL - SL	LMM (logit)	306	0.54	.590
START: BW - SW	LMM (logit)	306	-0.30	.763
START: SL – SW	LMM (logit)	306	-0.52	.605
DCR: BL – BW	LMM (logit)	306	-0.19	.852
DCR: BL - SL	LMM (logit)	306	-0.93	.352
DCR: BW - SW	LMM (logit)	306	-0.92	.357
DCR: SL – SW	LMM (logit)	306	-0.15	.880
CORR1: BL – BW	LMM (logit)	306	-0.71	.481
CORR1: BL - SL	LMM (logit)	308	-0.10	.918
CORR1: BW - SW	LMM (logit)	306	1.22	.223
CORR1: SL – SW	LMM (logit)	308	0.58	.563
CORN: BL – BW	LMM (logit)	306	-1.81	.072
CORN: BL - SL	LMM (logit)	310	-1.38	.168
CORN: BW - SW	LMM (logit)	306	2.48	.014*
CORN: SL – SW	LMM (logit)	309	1.95	.052
CORR2: BL – BW	LMM (logit)	306	-1.33	.185
CORR2: BL - SL	LMM (logit)	308	-0.73	.469
CORR2: BW - SW	LMM (logit)	306	2.90	.004**
CORR2: SL – SW	LMM (logit)	308	2.16	.031*
RAMP1: BL – BW	LMM (logit)	306	0.10	.917
RAMP1: BL - SL	LMM (logit)	308	0.28	.783
RAMP1: BW - SW	LMM (logit)	306	0.71	.478
RAMP1: SL - SW	LMM (logit)	308	0.47	.636
RAMP2: BL – BW	LMM (logit)	306	0.26	.797
RAMP2: BL - SL	LMM (logit)	307	2.32	.021*
RAMP2: BW - SW	LMM (logit)	306	0.19	.851
RAMP2: SL – SW	LMM (logit)	307	-1.95	.052
RAMP3: BL – BW	LMM (logit)	306	-0.04	.966
RAMP3: BL - SL	LMM (logit)	306	2.09	.037*
RAMP3: BW - SW	LMM (logit)	306	1.00	.316
RAMP3: SL – SW	LMM (logit)	306	-1.20	.229
RAMP4: BL – BW	LMM (logit)	308	-1.76	.080
RAMP4: BL - SL	LMM (logit)	306	1.29	.200
RAMP4: BW - SW	LMM (logit)	307	4.03	< .001***
RAMP4: SL - SW	LMM (logit)	306	0.96	.337
CIRC: BL – BW	LMM (logit)	309	-0.01	.994
CIRC: BL – SL	LMM (logit)	307	-0.4	.967
CIRC: BW – SW	LMM (logit)	309	-0.47	.641
CIRC: SL – SW	LMM (logit)	308	-0.45	.651
CENT: BL – BW	LMM (logit)	306	1.02	.310
CENT: BL – SL	LMM (logit)	306	1.22	.223
CENT: BW – SW	LMM (logit)	306	-0.98	.327
CENT: SL – SW	LMM (logit)	306	-1.19	.236

REST: BL – BW	LMM (logit)	306	0.19	.847
REST: BL – SL	LMM (logit)	306	-0.54	.590
REST: BW - SW	LMM (logit)	306	-0.27	.787
REST: SL - SW	LMM (logit)	306	0.46	.643
**zMCSF – Latency to first entry (zones)**				
START: BL – BW	LMM (oN)	290	0.27	.788
START: BL - SL	LMM (oN)	290	0.44	.664
START: BW - SW	LMM (oN)	288	0.12	.988
START: SL – SW	LMM (oN)	288	-0.16	.876
DCR: BL – BW	LMM (oN)	290	1.02	.308
DCR: BL - SL	LMM (oN)	290	0.61	.542
DCR: BW - SW	LMM (oN)	288	-1.10	.272
DCR: SL – SW	LMM (oN)	288	-0.67	.501
CORR1: BL – BW	LMM (oN)	288	0.80	.426
CORR1: BL - SL	LMM (oN)	296	0.29	.769
CORR1: BW - SW	LMM (oN)	288	-2.28	.024*
CORR1: SL – SW	LMM (oN)	296	-1.67	.097
CORN: BL – BW	LMM (oN)	290	2.15	.032*
CORN: BL - SL	LMM (oN)	301	1.44	.150
CORN: BW - SW	LMM (oN)	288	-2.17	.031*
CORN: SL – SW	LMM (oN)	300	-1.43	.154
CORR2: BL – BW	LMM (oN)	290	0.12	.903
CORR2: BL - SL	LMM (oN)	297	-0.55	.581
CORR2: BW - SW	LMM (oN)	288	-2.08	.039*
CORR2: SL – SW	LMM (oN)	296	-1.24	.217
RAMP1: BL – BW	LMM (oN)	290	-0.68	.496
RAMP1: BL - SL	LMM (oN)	297	-1.83	.069
RAMP1: BW - SW	LMM (oN)	288	-1.61	.108
RAMP1: SL - SW	LMM (oN)	296	-0.27	.789
RAMP2: BL – BW	LMM (oN)	290	-0.60	.547
RAMP2: BL - SL	LMM (oN)	293	-1.94	.053
RAMP2: BW - SW	LMM (oN)	288	-1.15	.251
RAMP2: SL – SW	LMM (oN)	291	0.29	.772
RAMP3: BL – BW	LMM (oN)	290	-0.21	.835
RAMP3: BL - SL	LMM (oN)	290	-1.35	.179
RAMP3: BW - SW	LMM (oN)	288	-0.59	.559
RAMP3: SL – SW	LMM (oN)	288	0.59	.554
RAMP4: BL – BW	LMM (oN)	294	-0.16	.873
RAMP4: BL - SL	LMM (oN)	290	-0.71	.478
RAMP4: BW - SW	LMM (oN)	292	-1.34	.180
RAMP4: SL - SW	LMM (oN)	288	-0.83	.410
CIRC: BL – BW	LMM (oN)	298	1.95	.052
CIRC: BL – SL	LMM (oN)	293	1.70	.090
CIRC: BW – SW	LMM (oN)	299	-1.73	.084
CIRC: SL – SW	LMM (oN)	295	-1.47	.143
CENT: BL – BW	LMM (oN)	288	-0.29	.771
CENT: BL – SL	LMM (oN)	288	-0.32	.750
CENT: BW – SW	LMM (oN)	288	-0.98	.328
CENT: SL – SW	LMM (oN)	288	-0.95	.343
REST: BL – BW	LMM (oN)	288	-0.29	.774
REST: BL – SL	LMM (oN)	288	-0.01	.989
REST: BW - SW	LMM (oN)	288	0.48	.634
REST: SL - SW	LMM (oN)	288	0.20	.839

SF-3: Statistical data of the significantly differentially expressed genes in bold and shy fish with different social experiences.

**Ensembl**	**Gene**	**baseMean**	**LFC**	**LFC_SE**	p_{val}	p_{adj}
**HB: SL – SW (downregulated)**						
ENSDARG00000103398	fabp1b.2	9.97580	-3.12e^{-06}	.001443	3.15e^{-06}	.0306
ENSDARG00000103716	si:busm1-194e12.11	39.9764	-3.11e^{-07}	.001443	6.93e^{-14}	< .001
ENSDARG00000092233	vtg1	7.49148	-6.82e^{-07}	.001443	2.65e^{-19}	< .001
**HB: BL – BW (upregulated)**						
ENSDARG00000114577	si:dkey-159n16.2	41.6153	2.52316	.646558	3.39e^{-06}	.0036
ENSDARG00000074547	si:ch211-240l19.8	21.4076	4.31221	1.097784	2.87e^{-06}	.0034
ENSDARG00000038384	th2	45.3254	2.58e^{-06}	.001443	3.06e^{-07}	.0010
ENSDARG00000045139	ca7	10.4512	4.38669	1.022823	8.26e^{-07}	.0019
ENSDARG00000090428	ctrb1	63.6734	3.85813	1.090510	1.16e^{-05}	.0093
ENSDARG00000051923	ccnb1	21.0396	2.15329	.601519	1.26e^{-05}	.0097
ENSDARG00000007276	ela3l	30.7927	4.45723	1.178378	4.50e^{-06}	.0044
ENSDARG00000079497	tcima	1001.168	.74411	.194955	5.16e^{-06}	.0058
ENSDARG00000108939	FQ378016.1	149.2342	5.12e^{-06}	.001443	7.01e^{-07}	.0019
ENSDARG00000104773	junbb	1332.444	1.12732	.231937	4.81e^{-08}	< .001
ENSDARG00000099195	ier2a	209.1977	1.20487	.411377	.00010	.0487
ENSDARG00000013856	amy2a	59.87223	4.38541	1.048393	8.65e^{-07}	.0019
ENSDARG00000075008	pask	27.61162	1.10010	.370873	.00010	.0487
ENSDARG00000060397	hhip	885.2998	.55514	.168520	3.81e^{-05}	.0238
ENSDARG00000074378	junba	202.3109	.87789	.224327	3.31e^{-06}	.0036
ENSDARG00000058471	plk1	28.34337	1.71818	.524452	3.64e^{-05}	.0233
ENSDARG00000061697	ca14	8.674063	3.75428	.883215	1.58e^{-06}	.0025
ENSDARG00000095796	si:dkey-87o1.2	7.919607	2.87708	.972471	9.84e^{-05}	.0487
ENSDARG00000079274	prss59.1	89.94166	4.10973	.995604	1.16e^{-06}	.0022
ENSDARG00000073742	prss59.2	93.33689	4.06627	.975937	9.67e^{-07}	.0020
ENSDARG00000031683	fosab	459.0697	1.65026	.401785	1.51e^{-06}	.0025
ENSDARG00000104107	nkx2.4b	35.3621	1.46e^{-06}	.001443	2.25e^{-29}	< .001
ENSDARG00000056765	ela2l	69.70546	3.71018	1.103459	2.12e^{-05}	.0144
ENSDARG00000056744	ela2	34.00906	2.24264	.772010	.00011	.0493
ENSDARG00000100020	pim1	1117.054	.44426	.099439	3.26e^{-07}	.0010
ENSDARG00000037421	egr1	4246.108	1.39118	.287517	5.37e^{-08}	< .001
ENSDARG00000055752	npas4a	2373.923	2.08054	.419573	2.73e^{-08}	< .001
ENSDARG00000055250	cntd2	30.65171	2.62734	.769371	2.08e^{-05}	.0143
ENSDARG00000096645	si:ch211-131k2.2	86.64902	4.42e^{-06}	.001443	8.44e^{-05}	.0467
ENSDARG00000068846	zgc:66024	9.001743	2.93778	1.00989	.00011	.0493
ENSDARG00000000796	nr4a1	137.5082	1.76833	.475301	7.06e^{-06}	.0061
ENSDARG00000029822	cel.2	12.40278	4.08896	1.03305	2.87e^{-06}	.0034
ENSDARG00000017490	cel.1	23.16914	5.78811	1.47173	2.52e^{-06}	.0034
ENSDARG00000058682	cd8b	14.68529	3.44788	.912050	5.62e^{-06}	.0050
ENSDARG00000056248	si:dkey-183i3.5	34.77394	2.05416	.576999	1.30e^{-05}	.0097
ENSDARG00000086881	ier2b	268.4214	1.26348	.264806	7.61e^{-08}	< .001
ENSDARG00000094055	si:dkey-88l16.3	10.07586	2.59750	.851849	7.57e^{-05}	.0435
ENSDARG00000068976	bsx	44.76955	3.55e^{-06}	.001443	1.71e^{-06}	.0026
ENSDARG00000045887	mmp30	4.491145	2.03e^{-06}	.001443	3.19e^{-05}	.0211
ENSDARG00000017314	cela1.6	121.253	3.89265	.989368	2.55e^{-06}	.0034
ENSDARG00000020298	btg2	583.6895	.81380	.265443	6.86e^{-05}	.0405
ENSDARG00000097513	CT573383.1	35.69544	4.00335	1.39249	9.25e^{-05}	.0487
ENSDARG00000045638	slc13a1	73.23511	1.59e^{-06}	.001443	1.82e^{-05}	.0132
ENSDARG00000061416	c2cd4a	36.23096	1.28e^{-05}	.001443	.00010	.0487
ENSDARG00000098739	h2af1al	45.33396	2.21960	.760912	.00010	.0487
**HB: BL – BW (downregulated)**						
ENSDARG00000098462	CU570782.1	10.2594	-2.29e^{-06}	.001443	6.55e^{-05}	.0397
ENSDARG00000001870	atp1a1a.4	13.30379	-3.30e^{-06}	.001443	8.53e^{-05}	.0467
ENSDARG00000096273	si:dkey-3n22.9	63.31436	-8.69e^{-07}	.001443	4.37e^{-06}	.0044
ENSDARG00000094550	BX649490.2	29.53691	-5.68e^{-08}	.001443	9.20e^{-06}	.0076

ENSDARG00000111856	BX649490.4	27.61877	-6.92e^{-08}	.001443	9.45e^{-05}	.0487
**HB: SL – BL (upregulated)**						
ENSDARG00000096187	si:dkey-21h14.10	4.678902	.00392	.041370	8.92e^{-05}	.0256
ENSDARG00000070314	cald1a	255.1406	.44331	.127597	2.07e^{-05}	.0102
ENSDARG00000106267	CR388042.1	35.03077	5.24287	.869431	9.57e^{-11}	< .001
ENSDARG00000035820	drd4b	8.123519	.01271	.044010	2.99e^{-05}	.0128
ENSDARG00000100738	osmr	38.80564	.00421	.041412	7.94e^{-05}	.0243
ENSDARG00000074320	KCNAB3	228.6036	.51040	.179798	.00014	.0338
ENSDARG00000100972	myh11b	5.909423	4.59848	1.304109	2.06e^{-05}	.0101
ENSDARG00000098749	tbcd	553.2857	.29519	.104841	.00018	.0398
ENSDARG00000021720	col7a1	48.52985	1.66307	.474471	1.64e^{-05}	.0092
ENSDARG00000001913	palmda	74.66797	.002508	.041210	4.32e^{-05}	.0159
ENSDARG00000016994	ssrp1b	983.9128	.33829	.081903	1.83e^{-06}	.0024
ENSDARG00000061629	ndufaf5	240.9738	.29807	.101821	.00011	.0298
ENSDARG00000096586	si:ch1073-110a20.3	7.755719	4.51644	1.237995	1.21e^{-05}	.0083
ENSDARG00000020028	cps1	11.81904	1.91559	.594821	4.73e^{-05}	.0164
ENSDARG00000102888	gpr39	12.16084	.00786	.042198	4.66e^{-05}	.0164
ENSDARG00000032838	si:dkey-206f10.1	3.230056	6.33595	2.637496	9.21e^{-05}	.0261
ENSDARG00000098779	BX547930.4	7.434990	.00233	.041194	8.25e^{-05}	.0249
ENSDARG00000069407	zgc:194990	109.3109	.71567	.261430	.00019	.0415
ENSDARG00000036156	fnbp1a	283.9691	.59804	.217282	.00019	.0413
ENSDARG00000088116	gstm.3	34.85850	2.24500	.612938	8.76e^{-06}	.0688
ENSDARG00000062487	si:dkey-6n6.1	78.40270	1.66594	.308191	2.76e^{-09}	< .001
ENSDARG00000110838	agbl4	75.61300	.60852	.176271	2.19e^{-05}	.0103
ENSDARG00000116896	TMEM233	9.426251	3.69603	.928925	4.48e^{-06}	.0044
ENSDARG00000022503	pkd2l1	12.70354	2.59294	.970767	.000216	.0420
ENSDARG00000058486	caps2	54.22949	.84579	.296854	.000161	.0365
ENSDARG00000010296	kcnh6b	16.03450	3.9770	1.097234	9.23e^{-06}	.0070
ENSDARG00000016088	rtn2a	60.25248	.84956	.236418	1.28e^{-05}	.0084
ENSDARG00000112977	CABZ01061591.1	187.6869	.42882	.162103	.00027	.0474
ENSDARG00000059202	tspan2b	69.85738	1.06775	.370554	.00013	.0317
ENSDARG00000029905	phyhd1	133.7733	.81975	.231780	1.50e^{-05}	.0088
ENSDARG00000005141	camkvb	2239.715	.59149	.220080	.00023	.0437
ENSDARG00000074808	megf6b	45.56442	1.33145	.490102	.00020	.0418
ENSDARG00000089645	si:ch1073-406l10.2	71.83379	.70730	.248980	.00016	.0360
ENSDARG00000008790	actr3b	300.7535	.69241	.207170	2.59e^{-05}	.0114
ENSDARG00000026165	col11a1a	78.69788	.90149	.333775	.00022	.0420
ENSDARG00000071685	slco5a1a	169.7608	.47029	.173953	.00022	.0420
ENSDARG00000090145	tmem240b	367.1843	.53664	.176716	8.47e^{-05}	.0250
ENSDARG00000094550	BX649490.2	29.53691	.00038	.041099	2.90e^{-06}	.0030
ENSDARG00000095798	BX666064.1	24.36383	2.88525	.998531	.00011	.0295
ENSDARG00000063180	dock3	1869.461	.53576	.183241	.00012	.0298
ENSDARG00000111856	BX649490.4	27.61877	.00039	.041099	2.14e^{-06}	.0026
ENSDARG00000071076	ldhbb	839.0020	.62288	.189858	3.84e^{-05}	.0148
ENSDARG00000104235	myo5c	103.0197	2.25147	.624744	1.09e^{-05}	.0077
ENSDARG00000032650	fuk	194.8692	.51119	.147328	2.03e^{-05}	.0102
ENSDARG00000103230	CABZ01079302.1	24.87903	3.54540	.766015	1.55e^{-07}	< .001
**HB: SL – BL (downregulated)**						
ENSDARG00000022165	mgst1.2	284.038	-.93278	.319116	.00011	.0295
ENSDARG00000038384	th2	45.3253	-6.41714	1.704624	4.54e^{-08}	< .001
ENSDARG00000017010	scamp2l	18.77712	-.02040	.048802	.00020	.0418
ENSDARG00000088087	kdm6ba	1696.720	-.46403	.135927	2.61e^{-05}	.0114
ENSDARG00000010332	zgc:56231	11.37156	-.00536	.041610	3.51e^{-05}	.0144
ENSDARG00000051923	ccnb1	21.03961	-.01098	.043257	3.13e^{-05}	.0132
ENSDARG00000089769	hapln1a	44.89787	-.01203	.043686	.00014	.0333
ENSDARG00000035018	thy1	82.86500	-.01171	.043552	.00011	.0295
ENSDARG00000005943	htra4	15.05444	-.00273	0,041232	1.24e^{-05}	.0083
ENSDARG00000079497	tcima	1001.168	-.63156	0,199658	5.77e^{-05}	.0189
ENSDARG00000108939	FQ378016.1	149.2342	-.00813	0,042288	5.52e^{-06}	.0048
ENSDARG00000070484	zgc:195001	58.24178	-.00812	0,042268	.00012	.0317

ENSDARG00000038133	zgc:113411	78.79128	-1.00418	0,333284	8.59e^{-05}	.0250
ENSDARG00000037782	sox8b	47.35481	-.02126	0,049479	.00026	.0464
ENSDARG00000087012	BX004816.2	18.04599	-.00669	0,041896	4.51e^{-05}	.0161
ENSDARG00000044511	etv5b	1142.309	-.94065	0,275892	2.24e^{-05}	.0103
ENSDARG00000055192	zgc:136930	7.717208	-.00252	0,041211	.00020	.0415
ENSDARG00000033231	mcm6l	8.914312	-.00574	0,041685	4.17e^{-05}	.0156
ENSDARG00000097559	cyp8b3	15.43430	-.00488	0,041525	2.77e^{-06}	.0030
ENSDARG00000100741	cdc20	30.96875	-.01199	0,043652	.00026	.0464
ENSDARG00000094449	BX511231.1	14.95514	-.00200	0,041168	.00014	.0338
ENSDARG00000002330	lhx8a	48.80534	-.00516	0,041564	.00022	.0420
ENSDARG00000076043	si:dkeyp-73d8.9	38.31173	-.00975	0,042798	5.21e^{-05}	.0178
ENSDARG00000069018	cyp7a1	9.777166	-.00379	0,041355	3.69e^{-05}	.0147
ENSDARG00000104907	ing2	215.8740	-.36566	0,124877	.00013	.0321
ENSDARG00000058471	plk1	28.34337	-2.32654	0,503943	1.50e^{-07}	.0003
ENSDARG00000075929	si:dkey-219c10.4	35.78369	-2.70998	0,807650	3.55e^{-05}	.0144
ENSDARG00000069763	etv5a	2830.509	-0.74446	0,217520	2.20e^{-05}	.0103
ENSDARG00000079964	dlx2a	105.7694	-2.32205	0,624742	1.48e^{-05}	.0088
ENSDARG00000061697	ca14	8.674063	-.00865	0,042429	7.56e^{-05}	.0234
ENSDARG00000093760	si:ch211-197h24.9	69.90477	-.02474	0,052808	.00016	.0365
ENSDARG00000043457	gapdh	393.0814	-.00875	0,042474	1.44e^{-05}	.0088
ENSDARG00000029259	zgc:136493	213.7953	-1.33699	0,262303	1.32e^{-08}	< .001
ENSDARG00000070656	si:ch211-69g19.2	44.87583	-.01210	0,043713	.00013	.0329
ENSDARG00000060862	atxn1b	2255.713	-.39883	0,152312	.00023	.0442
ENSDARG00000015543	s100a1	19.60989	-.00788	0,042205	1.76e^{-05}	.0096
ENSDARG00000021787	abcb5	20.48300	-.00445	0,041451	6.83e^{-05}	.0214
ENSDARG00000014039	si:dkeyp-93d12.1	6.454126	-.00871	0,042439	.00017	.0382
ENSDARG00000088989	si:dkey-241l7.5	60.03630	-.00269	0,041229	1.06e^{-09}	< .001
ENSDARG00000105335	si:dkey-241l7.2	34.39383	-.00143	0,041134	6.70e^{-07}	.0012
ENSDARG00000094752	rpe65b	66.73917	-1.09521	0,262732	1.30e^{-06}	.0019
ENSDARG00000002295	si:dkey-21p1.3	6.842278	-.00656	0,041858	6.16e^{-05}	.0196
ENSDARG00000010572	slc25a25a	79.52563	-.02183	0,050016	.00017	.0387
ENSDARG00000057433	st6galnac5b	168.9028	-.61126	0,177332	2.05e^{-05}	.0102
ENSDARG00000058865	endog	119.0462	-.60587	0,177204	2.40e^{-05}	.0109
ENSDARG00000078216	eps15	887.4424	-.42562	0,130966	4.43e^{-05}	.0160
ENSDARG00000100020	pim1	1117.054	-.52578	0,097541	2.50e^{-09}	< .001
ENSDARG00000103716	si:busm1-194e12.11	39.97644	-.00051	0,041101	3.82e^{-13}	< .001
ENSDARG00000015657	zgc:77112	39.23900	-.01116	0,043357	4.76e^{-06}	.0045
ENSDARG00000071021	papss2a	68.39562	-.01951	0,048238	1.40e^{-05}	.0088
ENSDARG00000040623	fosl2	315.2186	-.02192	0,050121	.00016	.0360
ENSDARG00000100558	slbp	101.0754	-.74040	0,236396	5.93e^{-05}	.0191
ENSDARG00000075421	pttg1	9.603928	-.00721	0,042016	.00024	.0448
ENSDARG00000037393	slc43a1a	7.290583	-.00595	0,041720	.00026	.0464
ENSDARG00000055752	npas4a	2373.923	-.33653	0,446595	8.48e^{-05}	.0250
ENSDARG00000002197	pygl	194.4050	-.02214	0,050379	5.39e^{-05}	.0181
ENSDARG00000055250	cntd2	30.65171	-.00789	0,042212	1.60e^{-05}	.0092
ENSDARG00000076659	cdca7b	15.20359	-.01268	0,043955	.00028	.0489
ENSDARG00000068846	zgc:66024	9.001743	-.00592	0,041728	6.11e^{-06}	.0051
ENSDARG00000039007	eno3	573.5022	-.05443	0,152594	.00011	.0295
ENSDARG00000000796	nr4a1	137.5082	-.01354	0,044377	.00024	.0448
ENSDARG00000006603	csrp1a	66.00428	-.54570	0,202350	.00024	.0443
ENSDARG00000054211	st8sia7.1	2.054069	-.00378	0,041353	2.00e^{-05}	.0102
ENSDARG00000101595	tgm1l2	24.06534	-.00198	0,041167	.00028	.0492
ENSDARG00000053395	cdkn2aipnl	336.8879	-.35778	0,132669	.00021	.0420
ENSDARG00000058682	cd8b	14.68529	-.00663	0,041890	6.42e^{-07}	.0012
ENSDARG00000044691	ppp1r3b	10.14181	-.00671	0,041895	.00014	.0338
ENSDARG00000025174	zgc:103482	32.46348	-.01382	0,044605	1.17e^{-06}	.0018
ENSDARG00000018757	klf5l	3.058990	-.00401	0,041381	.00018	.0394
ENSDARG00000117089	CKS2	21.87011	-.01652	0,046098	3.82e^{-05}	.0148
ENSDARG00000068732	spry4	443.2542	-.53375	0,204834	.00027	.0480
ENSDARG00000086881	ier2b	268.4214	-1.11077	0,267289	1.09e^{-06}	.0018

ENSDARG00000089802	akap1a	2.358948	-.00400	0,041384	$4.99e^{-06}$	.0045
ENSDARG00000068976	bsx	44.76955	-.00534	0,041601	.00021	.0420
ENSDARG00000071735	prlh2	46.78754	-.00494	0,041535	$2.47e^{-06}$	.0028
ENSDARG00000045306	slc51a	37.97154	-.01404	0,044667	.00011	.0295
ENSDARG00000045453	f13a1a.1	49.04331	-.00343	0,041304	.00022	.0427
ENSDARG00000038429	csrnp1b	474.4105	-.52803	0,166938	$5.56e^{-05}$	.0184
ENSDARG00000071662	si:rp71-36a1.3	27.97897	-.00121	0,041123	.00010	.0287
ENSDARG00000092233	vtg1	7.491476	-.00193	0,041164	$2.21e^{-37}$	< .001
ENSDARG00000022372	kng1	34.04275	-4.29655	0,606940	$9.91e^{-14}$	< .001
ENSDARG00000102493	ticam1	145.5738	-.67717	0,208460	$4.02e^{-05}$	.0153
ENSDARG00000002365	cers5	209.1414	-.59727	0,164092	$1.02e^{-05}$	.0075
ENSDARG00000053918	srfa	221.9119	-.46776	0,171538	.00021	.0420
ENSDARG00000103183	CABZ01034691.1	10.06589	-.00665	0,041896	$1.39e^{-06}$	.0019
ENSDARG00000079645	sc:d217	179.2094	-.01378	0,044561	$7.46e^{-06}$	.0061
ENSDARG00000114409	CR450686.6	33.19857	-.00676	0,041906	.00015	.0360
ENSDARG00000102414	myhz1.1	100.4280	-.00362	0,041334	$2.45e^{-06}$	.0028
ENSDARG00000098360	cyp19a1b	10665.86	-.00608	0,041749	.00027	.0474
ENSDARG00000101164	nansb	1.959533	-.00672	0,041894	.00021	.0420
ENSDARG00000104040	CABZ01046088.1	7.020808	-.01344	0,044325	.00020	.0418
**HB: SW – BW (upregulated)**						
ENSDARG00000088836	si:ch211-76m11.5	24.21246	.00059	.0115717	$6.47e^{-05}$	.0445
ENSDARG00000106267	CR388042.1	35.03077	6.16282	.9884124	$6.45e^{-11}$	< .001
ENSDARG00000114783	znf985	4.228667	.00045	.0115626	$3.62e^{-05}$	.0281
ENSDARG00000028912	si:dkey-10h3.2	14.98272	3.21717	.7314620	$4.52e^{-07}$	.0011
ENSDARG00000018077	rbp1.1	114.7749	1.63333	.3958970	$1.34e^{-06}$	.0029
ENSDARG00000014790	g3bp2	1418.021	1.26125	.3527051	$1.12e^{-05}$	.0123
ENSDARG00000102888	gpr39	12.16084	4.37053	.9151636	$1.08e^{-07}$	< .001
ENSDARG00000104107	nkx2.4b	35.36210	.00017	.0115517	$7.92e^{-26}$	< .001
ENSDARG00000075405	adck5	181.5564	.72712	.1535012	$8.49e^{-08}$	< .001
ENSDARG00000088898	caln1	443.8570	.61575	.2014710	$7.45e^{-05}$	.0460
ENSDARG00000093931	rflnb	350.3003	.47358	.1490874	$5.23e^{-05}$	.0382
ENSDARG00000102050	MCOLN3	22.21918	2.74090	.8918445	$6.30e^{-05}$	.0445
ENSDARG00000020811	efemp2b	225.5847	.87753	.2566612	$2.25e^{-05}$	.0193
ENSDARG00000019713	oatx	39.21077	.00021	.0115528	$1.59e^{-05}$	.0153
ENSDARG00000023609	AL845324.1	1102.719	.49986	.1343324	$7.32e^{-06}$	.0099
ENSDARG00000074808	megf6b	45.56442	1.70800	.4704327	$1.04e^{-05}$	.0123
ENSDARG00000094550	BX649490.2	29.53691	$2.95e^{-05}$	.0115500	$5.95e^{-06}$	.0099
ENSDARG00000111856	BX649490.4	27.61877	$2.96e^{-05}$	.0115500	$4.36e^{-06}$	.0087
ENSDARG00000045638	slc13a1	73.23511	.00021	.0115528	$6.66e^{-08}$	< .001
ENSDARG00000100332	CABZ01084942.1	12.94216	.00043	.0115614	$2.32e^{-05}$	.0193
ENSDARG00000100540	CABZ01114105.1	47.84465	2.82088	.7682446	$8.09e^{-06}$	.0103
**HB: SW – BW (downregulated)**						
ENSDARG00000098932	gigyf1a	514.4630	-.53129	.172418	$6.84e^{-05}$	.0448
ENSDARG00000023495	ift74	525.1150	-.00483	.013054	$7.07e^{-05}$	.0448
ENSDARG00000037805	lgals3bpa	55.73727	-.00069	.011580	$2.00e^{-05}$	.0179
ENSDARG00000037782	sox8b	47.35481	-.00181	.011754	$6.53e^{-06}$	.0099
ENSDARG00000055589	s100t	3.372115	-.00028	.011555	$6.94e^{-06}$	.0099
ENSDARG00000029259	zgc:136493	213.7953	-.00305	.012150	$7.20e^{-10}$	< .001
ENSDARG00000010478	hsp90aa1.1	50.37898	-.00083	.011593	$7.29e^{-06}$	.0099
ENSDARG00000088989	si:dkey-241l7.5	60.03630	-.00020	.011553	$7.93e^{-05}$	.0478
ENSDARG00000105214	agtpbp1	493.3345	-.30493	.100181	$6.99e^{-05}$	.0448
ENSDARG00000053481	entpd5a	25.66867	-.00087	.011597	$1.76e^{-05}$	.0163
ENSDARG00000031483	col9a1b	11.06776	-.00102	.011615	$4.93e^{-05}$	.0371
ENSDARG00000096327	cd164l2	14.68670	-.00098	.011611	$2.46e^{-07}$	< .001
ENSDARG00000096273	si:dkey-3n22.9	63.31436	-.00011	.011551	$7.38e^{-06}$	.0099
ENSDARG00000103586	si:dkey-65j6.2	22.36293	-.00141	.011674	$1.36e^{-05}$	.0142
ENSDARG00000054319	oxct1b	16.58725	-.00045	.011563	$8.13e^{-05}$	.0478
ENSDARG00000087633	si:dkey-11o18.5	118.2286	-.00040	.011560	$2.57e^{-08}$	< .001
ENSDARG00000089357	miga2	164.2681	-.00418	.012673	$1.42e^{-05}$	.0142

ENSDARG00000105511	BX248521.2	38.42651	-1.39769	.388132	$1.10e^{-05}$	.0123
ENSDARG00000022372	kng1	34.04275	-4.54008	.631777	$5.27e^{-14}$	< .001
ENSDARG00000104467	bglapl	16.29623	-.00086	.011595	$2.80e^{-05}$	.0225
**FBMB: SL – SW (downregulated)**						
ENSDARG00000103716	si:busm1-194e12.11	39.97644	$-3.13e^{-07}$	.001443	$3.12e^{-12}$	$9.11e^{-08}$
**FBMB: BL – BW (upregulated)**						
ENSDARG00000096243	ighv1-3	5.821612	$3.66e^{-07}$	.001443	$3.89e^{-09}$	< .001
ENSDARG00000061697	ca14	8.674063	3.04691	.763709	$2.84e^{-06}$	.0277
ENSDARG00000002347	cyp11a1	11.00258	$2.80e^{-07}$	.001443	$9.45e^{-08}$	.0014
**FBMB: SL – BL (upregulated)**						
ENSDARG00000106267	CR388042.1	35.03077	4.09343	.783176	$1.03e^{-08}$	< .001
ENSDARG00000100738	osmr	38.80564	3.80891	1.187676	$3.53e^{-05}$	.0290
ENSDARG00000020822	ift22	281.2796	.51977	.172667	$9.10e^{-05}$	.0498
ENSDARG00000097774	CR387996.1	3.468637	.00121	.019703	$7.24e^{-05}$	.0450
ENSDARG00000102888	gpr39	12.16084	.27457	.900024	$9.38e^{-06}$	.0135
ENSDARG00000007783	blk	53.89848	.25118	.663937	$2.32e^{-05}$	.0205
ENSDARG00000062487	si:dkey-6n6.1	78.40270	1.28260	.320015	$2.36e^{-06}$	.0068
ENSDARG00000076994	adgra2	29.16110	.97243	.293805	$3.76e^{-05}$	.0298
ENSDARG00000116896	TMEM233	9.426251	3.28898	.889375	$9.09e^{-06}$	.0135
ENSDARG00000010296	kcnh6b	16.03450	4.26442	1.242354	$2.03e^{-05}$	.0203
ENSDARG00000029905	phyhd1	133.7733	.78310	.228669	$2.21e^{-05}$	.0203
ENSDARG00000074808	megf6b	45.56442	1.72066	.475471	$1.01e^{-05}$	.0135
ENSDARG00000090219	wdr45	561.4647	.23736	.076515	$6.75e^{-05}$	.0431
ENSDARG00000008790	actr3b	300.7535	.64454	.215436	$9.01e^{-05}$	.0498
ENSDARG00000042753	cts12	49.14525	.93903	.308335	$7.73e^{-05}$	.0466
ENSDARG00000094550	BX649490.2	29.53691	$8.60e^{-05}$	.019649	$5.11e^{-06}$	.0098
ENSDARG00000104015	fgfr1bl	21.49476	2.36559	.573686	$1.54e^{-06}$	.0051
ENSDARG00000071339	borcs8	139.5423	.45237	.124500	$1.06e^{-05}$	.0135
ENSDARG00000111856	BX649490.4	27.61877	$8.60e^{-05}$	.019649	$4.77e^{-06}$	.0098
ENSDARG00000071076	ldhbb	839.0020	.77107	.186599	$1.44e^{-06}$	.0051
ENSDARG00000104235	myo5c	103.0197	2.39394	.622511	$4.31e^{-06}$	.0098
ENSDARG00000103230	CABZ01079302.1	24.87903	3.68635	.810422	$2.19e^{-07}$	.0013
ENSDARG00000100284	cd247	49.10169	1.32348	.372279	$1.37e^{-05}$	.0166
**FBMB: SL – BL (downregulated)**						
ENSDARG00000037805	lgals3bpa	55.73727	-.00190	.019782	$8.55e^{-05}$	.0491
ENSDARG00000037782	sox8b	47.35481	-.00488	.020532	$4.99e^{-05}$	.0359
ENSDARG00000075785	si:ch73-190m4.1	18.32180	-.00238	.019859	$1.70e^{-05}$	.0177
ENSDARG00000113332	CABZ01084501.2	54.06207	-.00270	.019918	$4.43e^{-05}$	.0340
ENSDARG00000029259	zgc:136493	213.7953	-1.02621	.275132	$7.02e^{-06}$	.0115
ENSDARG00000070604	zgc:162509	10.21273	-2.28453	.640860	$1.61e^{-05}$	.0176
ENSDARG00000057433	st6galnac5b	168.9028	-.01041	.023933	$2.18e^{-05}$	.0203
ENSDARG00000103716	si:busm1-194e12.11	39.97644	-.00012	.019650	$5.89e^{-13}$	< .001
ENSDARG00000105392	si:ch73-22o18.1	14.36229	-.00109	.019693	$4.95e^{-05}$	.0359
ENSDARG00000037403	hspa8b	2744.924	-.83804	.269041	$6.14e^{-05}$	.0415
ENSDARG00000037781	acss2	700.6046	-.21979	.062591	$1.55e^{-05}$	.0176
ENSDARG00000068732	spry4	443.2542	-.61872	.197473	$5.29e^{-05}$	.0368
ENSDARG00000002494	itgb6	42.03611	-.00160	.019744	$4.65e^{-06}$	.0098
ENSDARG00000075192	yme1l1a	370.0990	-.64764	.138760	$1.25e^{-07}$	< .001
ENSDARG00000022372	kng1	34.04275	-.00308	.020004	$4.06e^{-07}$	.0019
ENSDARG00000103183	CABZ01034691.1	10.06589	-3.5565	1.053463	$3.22e^{-05}$	.0274
ENSDARG00000079645	sc:d217	179.2094	-.00309	.020004	$5.74e^{-06}$	.0101
ENSDARG00000077357	lrrc61	129.8692	-.00868	.022533	$7.90e^{-05}$	.0466
ENSDARG00000005841	tnni2a.2	15.41668	-.00076	.019671	$6.35e^{-05}$	.0417
**FBMB: SW – BW (upregulated)**						
ENSDARG00000086615	CR846087.1	24.25384	1.80942	.584330	$6.62e^{-05}$	.0357
ENSDARG00000104561	znf1081	21.90228	1.89583	.645243	0.00011	.0481

ENSDARG00000106267	CR388042.1	35.03077	5.82921	.909595	$9.21e^{-12}$	< .001
ENSDARG00000003989	crhr1	326.8612	0.77404	.245134	$5.24e^{-05}$	.0324
ENSDARG00000096243	ighv1-3	5.821612	$8.89e^{-05}$	.016350	$1.61e^{-08}$	< .001
ENSDARG00000069940	ppap2d	846.4461	.38736	.113100	$2.21e^{-05}$	.0205
ENSDARG00000044341	chst7	1111.210	.35456	.111592	$5.22e^{-05}$	.0324
ENSDARG00000018077	rbp1.1	114.7749	1.77637	.392526	$2.40e^{-07}$	.0006
ENSDARG00000062661	abca4b	19.92965	2.46443	.801192	$6.43e^{-05}$	.0357
ENSDARG00000001913	palmda	74.66797	4.98298	1.639879	$4.38e^{-05}$	.0301
ENSDARG00000044501	viml	313.7329	1.87359	.447091	$1.03e^{-06}$	.0017
ENSDARG00000037256	si:ch211-145b13.5	168.8158	.00327	.016823	$3.41e^{-05}$	.0257
ENSDARG00000012574	slkb	394.8304	.45717	.147247	$6.54e^{-05}$	.0357
ENSDARG00000102888	gpr39	12.16084	4.91526	1.060189	$4.24e^{-07}$	.0010
ENSDARG00000075685	tlr7	563.9174	.62366	.158011	$2.88e^{-06}$	.0043
ENSDARG00000076994	adgra2	29.16110	.89893	.307523	0.00012	.0489
ENSDARG00000117011	ewsr1b	329.9797	1.43001	.454609	$5.39e^{-05}$	.0324
ENSDARG00000025504	gucy2f	9.359947	2.64989	.891730	$9.61e^{-05}$	.0454
ENSDARG00000093931	rflnb	350.3003	.76524	.142432	$3.32e^{-09}$	< .001
ENSDARG00000102356	scp2b	703.4846	.29361	.098682	0.00011	.0481
ENSDARG00000102050	MCOLN3	22.21918	3.32804	.853902	$3.43e^{-06}$	.0046
ENSDARG00000020811	efemp2b	225.5847	.89730	.257761	$1.75e^{-05}$	.0174
ENSDARG00000086746	prodha	1844.124	.56678	.159797	$1.58e^{-05}$	.0173
ENSDARG00000074808	megf6b	45.56442	1.59668	.473315	$2.56e^{-05}$	.0228
ENSDARG00000094550	BX649490.2	29.53691	$6.00e^{-05}$	.016350	$4.74e^{-06}$	.0060
ENSDARG00000111856	BX649490.4	27.61877	$5.90e^{-05}$	.016350	$5.42e^{-06}$	.0065
ENSDARG00000045827	lyrm5b	22.12814	4.42498	1.394725	$3.94e^{-05}$	.0279
ENSDARG00000002347	cyp11a1	11.00258	$6.68e^{-05}$	.016350	$1.54e^{-07}$	.0005
ENSDARG00000078707	sema7a	232.7492	.407375	.121829	$2.96e^{-05}$	.0241
**FBMB: SW – BW (downregulated)**						
ENSDARG00000054597	cnot6l	376.0056	-.00472	.017345	$6.67e^{-05}$	.0357
ENSDARG00000046090	dhrs11a	138.8515	-.00570	.017810	$8.70e^{-05}$	.0437
ENSDARG00000088753	cfap299	17.16989	-.26277	.293151	$7.10e^{-07}$	.0013
ENSDARG00000037805	lgals3bpa	55.73727	-.00140	.016437	$1.32e^{-05}$	.0152
ENSDARG00000113977	fthl29	15.98906	-.00093	.016388	$3.00e^{-05}$	.0241
ENSDARG00000101322	tfr1a	258.4248	-.00363	.016938	$1.81e^{-05}$	.0174
ENSDARG00000029259	zgc:136493	213.7953	-.36098	.270908	$2.36e^{-08}$	< .001
ENSDARG00000020084	tg	28.72713	-.00133	.016430	$1.04e^{-06}$	.0017
ENSDARG00000020114	slc20a1a	306.0312	-.00140	.016436	0.00011	.0481
ENSDARG00000100020	pim1	1117.054	-.33719	.104617	$5.11e^{-05}$	.0324
ENSDARG00000007693	nfkbiab	1082.677	-.00662	.018379	$3.14e^{-06}$	.0045
ENSDARG00000057644	adam8b	102.6395	-.00465	.017314	$7.99e^{-05}$	.0415
ENSDARG00000057206	nmt1b	95.62002	-.06368	.357218	$9.36e^{-05}$	.0451
ENSDARG00000086762	gsdmea	84.33024	-.00326	.016821	$3.87e^{-05}$	.0279
ENSDARG00000037551	pm20d1.1	38.18667	-.00075	.016375	$6.82e^{-07}$	.0013
ENSDARG00000095962	fhdc5	46.44994	-.00234	.016591	$9.31e^{-05}$	.0451
ENSDARG00000054319	oxct1b	16.58725	-.00093	.016389	$2.84e^{-05}$	.024
ENSDARG00000087633	si:dkey-11o18.5	118.2286	-.00080	.016379	$8.08e^{-09}$	< .001
ENSDARG00000060390	stk26	207.8554	-.69370	.208613	$3.10e^{-05}$	.0241
ENSDARG00000105511	BX248521.2	38.42651	-.00377	.016996	$5.26e^{-07}$	.0012
ENSDARG00000024295	slc11a2	241.8629	-.41154	.140339	0.00011	.0481
ENSDARG00000104047	si:ch211-262i1.3	205.5569	-.00324	.016816	0.00011	.0481
ENSDARG00000002494	itgb6	42.03611	-.00123	.016418	$4.01e^{-09}$	< .001
ENSDARG00000091762	zbtb40	95.17587	-.00683	.018480	$8.10e^{-05}$	.0415
ENSDARG00000087186	si:ch211-232b12.5	899.7481	-.55184	.176480	$4.72e^{-05}$	.0316
ENSDARG00000013477	gata1a	121.8890	-.00332	.016840	$5.58e^{-05}$	.0328
ENSDARG00000026098	si:dkey-13a21.4	15.25367	-.00125	.016419	0.00012	.0489
ENSDARG00000022372	kng1	34.04275	-.95705	.598320	$1.48e^{-12}$	< .001
ENSDARG00000101362	mibp	1026.365	-.00198	.016524	$1.79e^{-05}$	.0174

References

1. Chen, C.-H., Poss, K., & Hughes, H. (2017). Adult Zebrafish p-Chip Implantation Protocol. *PharmaSeq*.

2. Thörnqvist PO, McCarrick S, Ericsson M, Roman E, Winberg S. Bold zebrafish (*Danio rerio*) express higher levels of delta opioid and dopamine D2 receptors in the brain compared to shy fish. Behav Brain Res 2019, 359: 927–934.
